# Combinatorial activities of SHORT VEGETATIVE PHASE and FLOWERING LOCUS C define distinct modes of flowering regulation in *Arabidopsis*

**DOI:** 10.1186/s13059-015-0597-1

**Published:** 2015-02-11

**Authors:** Julieta L Mateos, Pedro Madrigal, Kenichi Tsuda, Vimal Rawat, René Richter, Maida Romera-Branchat, Fabio Fornara, Korbinian Schneeberger, Paweł Krajewski, George Coupland

**Affiliations:** Department of Plant Developmental Biology, Max Planck Institute for Plant Breeding Research, D-50829 Cologne, Germany; Department of Biometry and Bioinformatics, Institute of Plant Genetics, Polish Academy of Sciences, 60-479 Poznań, Poland; Department of Plant Microbe Interactions, Max Planck Institute for Plant Breeding Research, D-50829 Cologne, Germany; Department of Biosciences, University of Milan, 20133 Milan, Italy; Present address: Fundación Instituto Leloir, Instituto de Investigaciones Bioquímicas de Buenos Aires - CONICET, C1405BWE, Buenos Aires, Argentina; Present address: Wellcome Trust Sanger Institute, Wellcome Trust Genome Campus, Hinxton, Cambridge, CB10 1SA UK; Present address: Wellcome Trust - Medical Research Council Cambridge Stem Cell Institute, Anne McLaren Laboratory for Regenerative Medicine, Department of Surgery, University of Cambridge, CB2 0SZ Cambridge, UK

## Abstract

**Background:**

The initiation of flowering is an important developmental transition as it marks the beginning of the reproductive phase in plants. The MADS-box transcription factors (TFs) FLOWERING LOCUS C (FLC) and SHORT VEGETATIVE PHASE (SVP) form a complex to repress the expression of genes that initiate flowering in Arabidopsis. Both TFs play a central role in the regulatory network by conferring seasonal patterns of flowering. However, their interdependence and biological relevance when acting as a complex have not been extensively studied.

**Results:**

We characterized the effects of both TFs individually and as a complex on flowering initiation using transcriptome profiling and DNA-binding occupancy. We find four major clusters regulating transcriptional responses, and that DNA binding scenarios are highly affected by the presence of the cognate partner. Remarkably, we identify genes whose regulation depends exclusively on simultaneous action of both proteins, thus distinguishing between the specificity of the SVP:FLC complex and that of each TF acting individually. The downstream targets of the SVP:FLC complex include a higher proportion of genes regulating floral induction, whereas those bound by either TF independently are biased towards floral development. Many genes involved in gibberellin-related processes are bound by the SVP:FLC complex, suggesting that direct regulation of gibberellin metabolism by FLC and SVP contributes to their effects on flowering.

**Conclusions:**

The regulatory codes controlled by SVP and FLC were deciphered at the genome-wide level revealing substantial flexibility based on dependent and independent DNA binding that may contribute to variation and robustness in the regulation of flowering.

**Electronic supplementary material:**

The online version of this article (doi:10.1186/s13059-015-0597-1) contains supplementary material, which is available to authorized users.

## Background

Developmental programs of multicellular organisms require the establishment of defined temporal and spatial patterns of gene expression. In higher plants, the initiation of flowering is the first step in reproductive development, and is controlled by a complex regulatory network that converges on the transcription of a small number of floral integrator genes [1,2]. This network primarily consists of transcription factors (TFs) that bind to *cis*-regulatory elements controlling gene transcription. Identifying the individual binding sites of TFs and their precise modes of regulation enables definition of such networks. The genome-wide binding sites of several TFs involved in flowering-time control and floral development were described based on the results of chromatin immunoprecipitation (ChIP) focused on single TFs [3-10]. However, many TFs act in protein complexes, and both their activity and the sites to which they bind are likely to be strongly modulated by the presence or absence of other members of the complex [8,11,12]. Here, we examined the individual and combined activities of the MADS-box TFs SHORT VEGETATIVE PHASE (SVP) and FLOWERING LOCUS C (FLC), which, as flowering repressors that control the effect of environmental cues on floral induction, fulfil key functions within the network controlling flowering in *Arabidopsis thaliana*.

Over 2,000 TFs are encoded in the *A. thaliana* genome and these were classified into 58 families [13], many of which contribute to developmental programs. Among these TFs, the MADS domain family represents a group conserved in nearly all eukaryotes, but greatly expanded in land plants [14]. MADS domain TFs in plants are heavily involved in controlling different stages of flowering, including the floral transition [15-18] and determination of floral organ identity [7,19-23] in which interacting MADS-box proteins form multimeric complexes that configure the ABC model [24]. According to this model different combinations of MADS-box TFs act in transcriptional complexes to specify distinct floral organ identities, which emphasises the significance of MADS protein complexes in conferring specificity.

Two members of the MADS-box family, FLC and SVP, enhance the flowering response to seasonal cues by repressing flowering under non-inductive conditions. These functions were defined by genetic analysis that placed FLC in the autonomous and vernalisation pathways [16,18]. *FLC* represses flowering but its mRNA level is reduced by exposure to winter cold (vernalisation), allowing flowering to proceed [16,18]. Similarly, SVP strongly delays flowering under non-inductive short days (SDs) but its transcription declines in the inflorescence meristem under inductive long days (LDs) [25,26]. *SVP* mRNA reappears shortly after in floral primordia, allowing this TF to participate in flower development [27,28]. The abundance and activity of SVP protein are also reduced under high ambient temperatures that promote flowering [8,15,29,30].

The inhibition of flowering caused by FLC and SVP is, at least in part, due to repression of the floral integrator genes *FLOWERING LOCUS T* (*FT*) and *SUPPRESSOR OF OVEREXPRESSION OF CONSTANS1* (*SOC1*) [12,30]. While *FT* is expressed in the leaves during floral induction, the major site of action for *SOC1* is in the shoot apical meristem. FLC acts in both organs to repress expression of these genes and delay flowering [12,31]. Similarly, genetic analysis, gene expression data and transgenic plants implicate SVP protein as acting in both tissues [31,32]. Mutations in either *FLC* or *SVP* lead to early flowering and increased levels of *FT* and *SOC1* mRNAs [15,16,18]. Notably, the double mutant results in an even earlier flowering phenotype, indicating that *SVP* and *FLC* have partially redundant functions during floral repression [12,33].

Consistent with their shared activity in repressing flowering and the capacity of many other MADS-box TFs to form transcriptional complexes, SVP and FLC proteins directly interact [12,33]. In agreement with this conclusion, each TF binds to similar regions of *FT* and *SOC1* [3,4,9,31,34]. In addition, these proteins form high order molecular complexes with other MADS-box proteins, including MADS AFFECTING FLOWERING 3 (MAF3) and FLOWERING LOCUS M (FLM), which belong to the same subclade of the family as FLC [8,27,35,36]. Functional analyses of SVP-FLM complex revealed the participation of SVP in the regulation of flowering response to ambient temperature [8,29]. Furthermore, a high throughput yeast two hybrid interaction map of *A. thaliana* MADS-box proteins also reported, among others, APETALA1 (AP1), SOC1, and SEPALLATA 3 (SEP3) as components of the SVP interactome [35], and these interactions contribute to flower meristem identity [26,27,37].

The activities of SVP and FLC were studied by ChIP PCR on individual target genes, and their genome-wide binding maps were characterized separately by ChIP followed by sequencing (ChIP-seq) or ChIP-on-chip approaches [3,4,9]. This analysis led to the identification of pathways in which each TF participates throughout development. However, no attempt has been made to decode their combined activity across the genome. More generally, TF binding sites are usually compared in separate experiments. However, defining binding sites of TF 'A' in the mutant background of its partner 'B' and *vice versa* has the potential to identify *bona fide* dependencies and to elucidate how both factors act in a combinatorial fashion to regulate transcription.

Here, we studied the combinatorial activity of the TFs FLC and SVP at a genome-wide level by combining ChIP-seq and transcriptome gene expression profiling on experiments performed on different genetic backgrounds and tissues. Through this comprehensive approach we identified direct targets of SVP in the presence and absence of its partner FLC. Reciprocally, FLC ChIP-seq was performed in wild-type and the *svp* mutant. Our study shows that the formation of the FLC:SVP complex substantially influences occupancy of each factor at specific binding sites. The findings demonstrate that complex formation is indeed essential for DNA binding to a subset of genes, leading to the identification of novel *cis*-regulatory elements that may contribute to the recruitment of the complex. The distinct spatial roles of SVP and FLC are emphasised by the marked differences in observed gene expression profiles between leaves and apices. Thus, by describing the genome-wide effects of two MADS-box proteins capable of forming a multimeric complex we show how their interactions generate diverse patterns of co-regulation.

## Results

### Transcriptional network controlled by FLC and SVP in leaves and apices

FLC and SVP delay flowering by repressing the expression of target genes such as *FT* and *SOC1* in the leaves and apices, respectively [30,31,34], and were reported to participate in the same protein complex [12]. SVP and FLC influence flowering time through various genetic pathways, but a specific function for their interaction remains unknown. We addressed this issue at the level of gene regulation in leaves and apices by performing genome-wide transcriptome analyses in single and double mutants of *SVP* and *FLC* under SDs. All plants carried a functional *FRIGIDA* (*FRI*) allele to ensure high levels of *FLC* transcription [38]. The transcriptome profiles of each single and double mutant were compared to wild-type *SVP FLC FRI* (WT) and differentially expressed genes (DEGs) were selected for subsequent analysis (Additional file 1).

**Figure 1:**
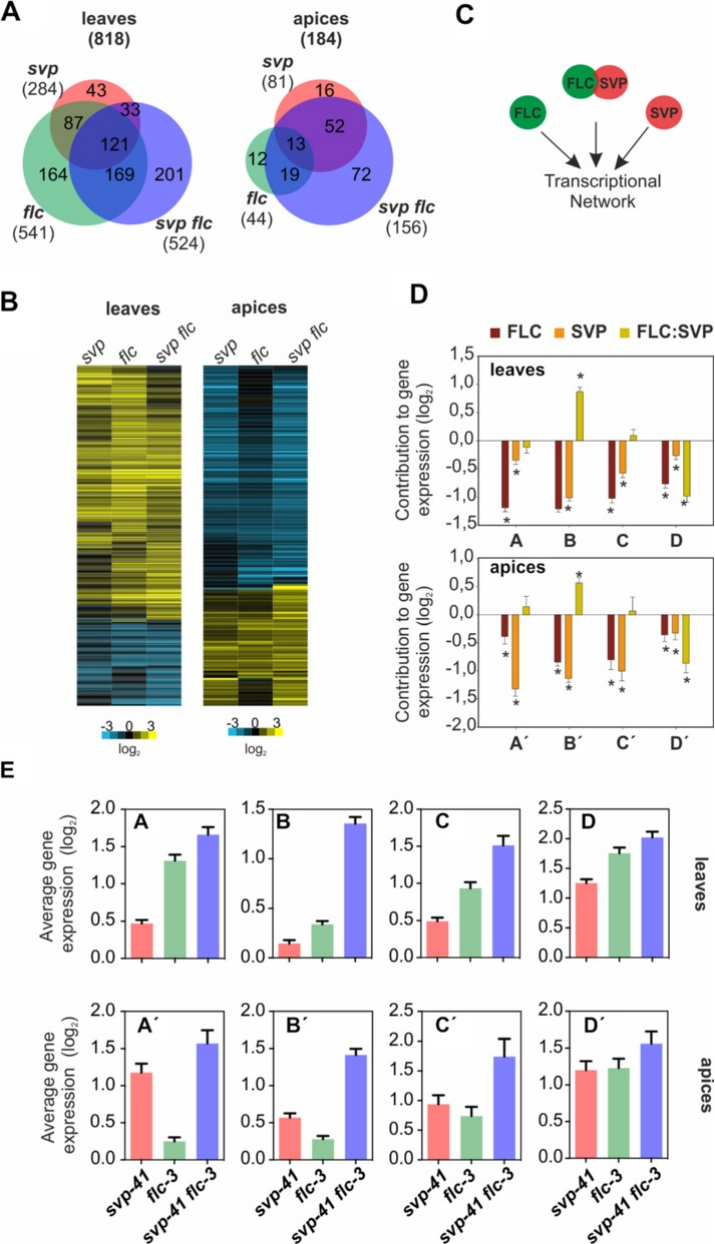
**Characterization of the transcriptional network controlled by FLC and SVP in leaves and apices. (A)** Genes differentially expressed in *svp-41* (pink), *flc-3* (green) and *svp-41 flc-3* (light-blue) loss of function mutants compared to wild-type *SVP FLC FRI* in leaves and apices. Plants were grown for 2 weeks under SD conditions, and leaves and apices were collected 8 h after dawn (Zeitgeber 8 (ZT8)). Only genes with fold-change above 2 and q-value <0.01 were selected as differentially expressed. **(B)** Transcriptional profile comparisons from (A) represented as a heatmap to highlight up-regulated (yellow) and down-regulated (blue) genes. Expression change is represented in log_2_ scale. **(C)** Schema of the transcriptional network under study. **(D)** Statistical analysis of signalling allocation from identified clusters. **A**, **B**, **C**, **D** and **A’**, **B’**, **C’**, **D’** denote the different expression clusters found in leaves and apices, respectively. Asterisks denote *P*-value <0.01. **(E)** Average change in expression level relative to wild-type for genes located in the four clusters represented in **(D)**. For each cluster, the average expression level in each mutant genotype is represented. Colour code is as in **(A)**. Cluster A, 33 genes; cluster B, 29 genes; cluster C, 18 genes; cluster D, 62 genes; cluster A’, 8 genes; cluster B’, 11 genes; cluster C’, 5 genes; cluster D’, 6 genes. Mean values are accompanied by standard error.

DEGs differed between apices and leaves (Figure 1A). Overall, more genes changed in expression in leaves (818 genes) than in apices (184 genes), suggesting that SVP and FLC have broader roles in regulating gene expression in leaves. Furthermore, tissue-specific differences in the contributions of FLC and SVP were observed. While FLC regulated approximately twice as many genes as SVP in leaves, this relationship was reversed in apices (Figure 1A).

Between 70 and 90% of DEGs in leaves showed increased transcript levels in mutants, in agreement with FLC and SVP acting predominantly as transcriptional repressors. However, in apices only 33 to 40% of DEGs increased in expression (Figure 1B; Additional file 1), suggesting that many of the DEGs in the apical samples are indirectly regulated by FLC and SVP, or that these TFs may act more frequently as transcriptional activators in the apex. Among the genes that were up- or down-regulated in leaves or apices, only 10 to 15% were shared between the three mutant genotypes (121 in leaves and 13 in apices, Figure 1A) and these behaved similarly in single and double mutants (Figure 1B; Additional file 1). Moreover, DEGs identified only in double mutants were usually affected to a small degree in each single mutant (less than two-fold compared with WT and therefore below the cut-off to call a DEG; Figure 1B; Additional file 1), suggesting an additive role of both TFs in the regulation of these genes.

The roles of FLC and SVP in controlling gene expression during the transition to flowering were then evaluated. Flowering of *A. thaliana* is induced by exposure to LDs, and five LDs were sufficient to commit plants to flower [39]. Therefore, to identify genes that respond early in the flowering process, before commitment, another set of expression profiling experiments was performed on the same genotypes two days after transferring plants from SDs to LDs. SVP and FLC were required for the differential expression of 30 to 40% of the DEGs identified during transition from SDs to LDs in WT (Additional files 2 and 3). Furthermore, only 20% of DEGs in WT were shared between leaves and apices upon transition to LDs, indicating that the processes occurring during induction of flowering differ between these tissues (Additional file 3).

Taken together, the gene expression profiling experiments revealed the signalling network defined by the two TFs and showed that they have differential yet partially redundant contributions in leaves and apices.

### Four patterns defined by FLC and SVP govern gene regulation in leaves and apices

Single mutations in *FLC* or *SVP* partially suppress the late-flowering phenotype of *SVP FLC FRI* while FLC and SVP physically interact, suggesting the resulting complex has a significant role in the repression of flowering [12,30]. If this protein complex were responsible for the full transcriptional response of SVP and FLC, single and double mutants would be expected to show similar transcriptome changes. However, our genome-wide transcriptome analysis demonstrated that transcriptional regulation by FLC and SVP cannot be explained in this way (Figure 1A,B), but rather is defined as the activity of the complex plus the activities of the individual TFs (Figure 1C).

To understand modes of gene regulation by FLC and SVP, signalling allocation analysis was performed [40,41]. A detailed description of the basis of this analysis is provided in Additional files 4 and 5. A linear model containing FLC, SVP and FLC:SVP as fixed terms explaining modes of transcriptional regulation was used to estimate the contribution to gene expression levels of FLC and SVP as single factors and of the FLC:SVP interaction (Figure 1D). The log_2_ expression values were used as the input data set for the analysis. Negative values for the fixed terms represent positive contributions to gene expression. So, if FLC:SVP took a negative value while FLC and SVP terms were zero, this was interpreted as the FLC:SVP complex having a role in gene expression whereas the single factors do not contribute. If the FLC:SVP term was zero, and FLC and SVP terms were negative, FLC and SVP were assumed to act independently with no action of the complex. Instead, a positive value for FLC:SVP and negative values for single proteins indicated that they function redundantly to repress gene expression, again with no contribution of the complex. All the genes up-regulated in the mutants compared with WT were used for this analysis. However, genes whose expression changes were larger in single mutants than the double mutant were excluded, because in this case gene regulation cannot be explained by transcriptional repression functions of FLC, SVP and the FLC:SVP complex but rather additional factors must be postulated to explain the data (Additional file 6). Nevertheless, we show transcript levels of genes with expression patterns only affected by either of the two TFs (Figure 1A) in Additional file 7.

Genome-wide modes of gene regulation by FLC and SVP were identified and classified into four representative clusters in leaves and apices (clusters A to D in leaves and A’ to D’ in apices in Figure 1D,E; Additional files 5 and 8). The clusters are based on the contribution of the terms of the linear model to gene expression (Additional file 4). Under SDs, 199 genes were up-regulated in leaves and 40 in apices. Of these upregulated genes, around 75% were included in the four clusters of interest (Additional file 8). In leaves, cluster A is mainly reliant on FLC (Figure 1D; Additional files 5 and 8). Consistently, this cluster includes genes showing significant up-regulation in *flc-3* but less in *svp-41* and a similar level of up-regulation in the *svp-41 flc-3* double mutant. Thus, genes in cluster A are regulated by FLC while the contribution of SVP or FLC:SVP complex to their regulation is very limited (Figure 1D,E; Additional file 8). Interestingly, in apices an analogous cluster was not detected, but instead an SVP-dependent cluster was identified (cluster A’; Figure 1D; Additional files 5 and 8). No common genes were found between clusters A and A’, strengthening the idea that these TFs control different genes as individual TFs in different tissues. Thus, when considering genes regulated individually by FLC or SVP during vegetative development, repression in leaves is mostly controlled by FLC while SVP plays the major role in apices, although we observe minor contributions of SVP in leaves and FLC in apices (Figure 1D; Additional files 5 and 8).

Unlike clusters A and A’, the expression patterns of clusters B and D and B’ and D’ were similar in both leaves and apices. Expression of genes in clusters B and B’ was greatly affected in the double mutant while much less in single mutants (Figure 1E), suggesting that SVP and FLC can compensate for each other in the regulation of these genes. This conclusion is statistically confirmed by a significant positive coefficient for FLC:SVP and negative coefficients for FLC and SVP (Figure 1D; Additional file 5). Cluster B in leaves included genes regulating leaf cell size at an early developmental stage, such as *ROTUNDIFOLIA*-like [42] and *TEOSINTE BRANCHED1/CYCLOIDEA/PROLIFERATING CELL FACTOR 9* (*TCP9*) [43]. Leaf shape changes were previously reported in *svp* and *flc* mutants [15,44], and *FLC* was found to have a direct role in leaf development [44]. Notably, *TCP* genes were also overrepresented among direct targets of other MADS-box proteins such as SEP3 and AP1 [7,20,45].

In clusters C and C’ (Figure 1D,E; Additional file 5), FLC and SVP individually contribute to gene expression in an additive manner as no significant effect of FLC:SVP was detected, whereas significant negative coefficients for FLC and SVP were observed (Figure 1D). This analysis indicates that FLC and SVP act independently to regulate these genes. Interestingly, cluster C’ in apices includes *GIBBERELLIN 20 OXIDASE 2* (*GA20OX2*), which encodes an enzyme involved in gibberellin (GA) biosynthesis and was recently shown to be repressed by *SVP* at the shoot apex [32]. Here FLC was also found to regulate expression of *GA20OX2* independently of SVP (Additional file 9), suggesting that both TFs actively control bioactive levels of GA through this enzyme.

Taken together, the genome-wide regulatory input of FLC and SVP revealed different quantitative contributions of the individual MADS-box TFs in regulating gene expression in different tissues, as well as considerable flexibility and diversity in their transcriptional responses, leading to independent (clusters A and A’), compensatory (clusters B and B’) or additive (clusters C and C’) modes of co-regulation.

### The FLC:SVP complex participates in transcriptional regulation

The extent to which FLC:SVP regulates gene expression was then addressed. Clusters D (62 genes) and D’ (6 genes) exhibited negative interaction coefficients for FLC:SVP, indicating that FLC and SVP act in the same pathway and consistent with the proteins acting in a heterodimer to regulate the expression of these genes. We propose that their regulation could depend on the protein complex (Figure 1D; Additional files 5 and 8). Similarly, the effect of mutating one TF on the expression of these genes was similar to the effect of mutating both (Figure 1E). This is particularly evident for cluster D’, whereas in cluster D a broader diversity in coefficient terms was observed (Additional file 5) and the average expression of the cluster indicates that FLC and SVP do not contribute equally but that FLC can work to some extent alone (Figure 1D).

Genes in these clusters that are involved in hormone-regulated processes or cold response were analysed in more detail. The *JASMONATE ZIM-domain* (*JAZ*) genes (*JAZ 3*, *5*, *6* and *8*), which contribute to the jasmonate signalling pathway [46,47], were regulated by FLC:SVP under SDs. The same association was shown by *DARK INDUCIBLE 10* (*DIN10*), also known as RAFFINOSE SYNTHASE, which encodes a glycosyl hydrolase associated with cold response, as well as light and sucrose stimulus. In *Arabidopsis*, *DIN10* transcript levels respond to cold treatments predicted to reduce *FLC* expression [48]. This observation was confirmed by measuring the level of *DIN10* mRNA by independent real-time quantitative PCR (qRT-PCR) in leaves of *svp*, *flc* and *svp flc* mutants (Additional file 10). The *din10* mutant also showed a weak but significant early-flowering phenotype, which can be correlated with increased levels of *FT* mRNA (Additional file 10).

Cluster D, whose expression is governed by FLC:SVP, contains 43% of the up-regulated genes in leaves under SDs while only 20% of the genes are present in cluster B, the compensation cluster. The signalling allocation analysis was repeated for plants shifted from SDs to LDs to evaluate the effect of an environmental stimulus that induces flowering. Interestingly, the proportion of genes controlled by the complex differed under SDs and LDs (Additional file 8). Contrary to what was found under SDs, cluster B contained almost 68% of the genes under LDs, constituting the main mode of transcriptional regulation under this condition (Additional file 8), whereas cluster D contained only 11% of the genes. This decrease in the proportion of FLC:SVP-dependent genes in leaves upon shift to LDs is in agreement with a weaker interaction between FLC and SVP detected in the leaves of 3- and 7-day-old LD grown seedlings [12], suggesting that FLC:SVP complex might not form or regulate gene expression under these conditions.

Overall, by separately analysing single and double mutants the contribution of the complex to gene expression was determined. These results indicate that FLC:SVP regulates around one third of the genes repressed by these proteins in the leaves under SDs but many fewer under LDs.

### SVP influences the genome-wide binding scenario of FLC and *vice versa*

The extent to which the described expression patterns can be explained by direct binding of FLC, SVP or FLC:SVP was determined by performing ChIP-seq to identify the genome-wide binding sites for each TF in the presence and absence of the other. Binding of SVP:GFP was studied in the above-ground tissue of seedlings using an anti-green fluorescent protein (GFP) antibody in the genotypes *pSVP:SVP:GFP svp-41 FLC FRI* (hereafter SVP:GFP in WT) and *pSVP:SVP:GFP svp-41 flc-3 FRI* (hereafter SVP in *flc-3*) (Additional file 11), allowing the influence of FLC on SVP:GFP binding to be determined. Similarly, polyclonal FLC antibody [49] was used for ChIP-seq in *SVP FLC FRI* (FLC in WT) and *svp-41 FLC FRI* backgrounds (FLC in *svp-41*). Each ChIP-seq was done in triplicate. Irreproducible discovery rate (IDR) analysis was performed and only the peaks that were reproducible at an IDR ≤ 0.05 were selected for further processing (Materials and methods; Additional file 12).

**Figure 2:**
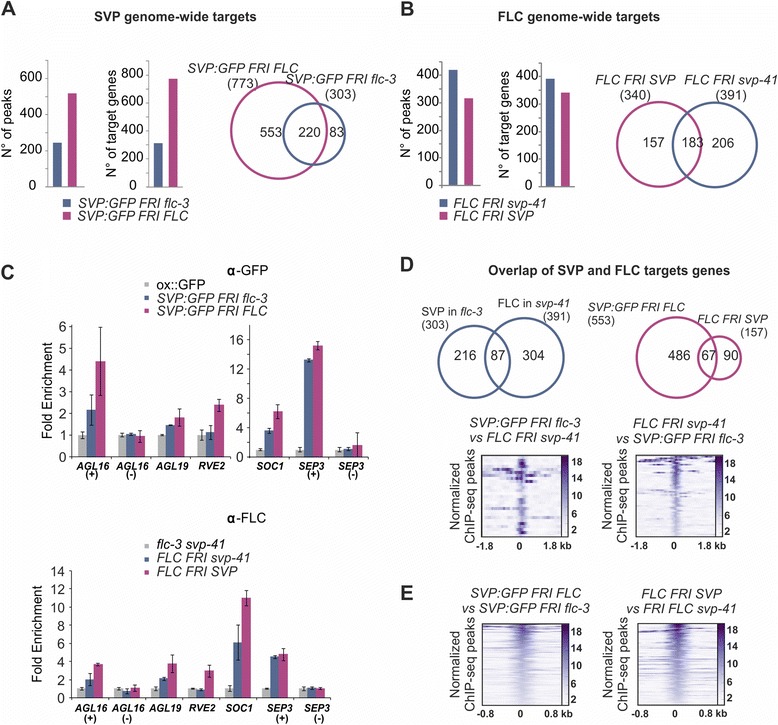
**Absence of cognate partner in the SVP-FLC complex influences genome-wide binding events of individual transcription factors. (A)** Number of significantly read-enriched peaks and their corresponding genes (left) for SVP binding in FLC (violet) and in *flc-3* mutant genotype (blue). Overlap among target genes for both genotypes (right). **(B)** Number of significantly enriched peaks and their corresponding genes (left) for FLC binding in SVP (purple) and in *svp-41* mutant genotype (blue) (left). Overlap among target genes for both genotypes (right). **(C)** Validation of selected targets. For each target, fold-enrichment relative to its input is shown. Plus signs indicate primers flanking predicted binding site; minus signs indicate primers not flanking predicted binding site used as negative control. Mean values are accompanied by standard error. **(D)** Top left: Venn diagram displaying the number of overlapping targets highly enriched for SVP binding in *flc-3* mutant and highly enriched for FLC binding in *svp-41* mutant ChIPs. Shared targets (total 87) are shown in the overlap area. Top right: Venn diagram displaying the number of overlapping targets highly enriched in ChIP for SVP binding only in WT and highly enriched for FLC binding only in WT ChIPs. Bottom: heat maps for a selection of 84 peaks for SVP binding in *flc-3* mutant found to overlap the peaks for FLC binding in *svp-41* mutant. Normalized binding signals are visualized 1.75 kb around each peak summit. The binding regions were sorted according to their maximum median value for FLC in the *svp-41* mutant. **(E)** Heatmap of normalized binding signals for 148 peak summits corresponding to SVP binding in FLC WT that overlap the peaks found for SVP binding in *flc-3* mutant (left), and for 170 peak summits corresponding to FLC binding in SVP WT and overlapping peak regions for FLC binding in *svp-41* mutant (right). In both cases, results were sorted according to the maximum median values and visualized for genomic regions 750 bp around peak summits.

In WT, SVP:GFP binding was detected at 523 unique genomic regions that were assigned to 773 neighbouring genes (peaks reside 3 kb upstream of the transcription start site or 1 kb downstream of the transcription end site) (Figure 2A; Additional file 13). On the other hand, in the *flc-3* mutant 264 peaks were called for SVP:GFP and annotated to 303 genes (Figure 2A; Additional file 13). Among these genes, 220 were bound in both genotypes, indicating that SVP:GFP binds to these genes regardless of the presence or absence of FLC. Conversely, 83 genes were identified only when FLC was absent, whereas 553 genes were bound only in the presence of FLC (Figure 2A). The proportion of SVP:GFP target genes found only in WT or *flc-3* differed significantly between the two genotypes (*P* <2.2e-16, Chi-square test). Thus, overall the presence of FLC increased the number of SVP:GFP targets, but targets only bound in the absence of FLC were also detected. Binding to the flowering-related genes *SEP3*, *SOC1* and *SCHLAFMUTZE* (*SMZ*), which were previously shown to be targets of SVP [4,9,11,12], did not depend on the presence of FLC. However, *TEMPRANILLO 1* (*TEM1*), *CONSTANS-LIKE 1* (*COL1*) and *CONSTANS-LIKE 4* (*COL4*) were exclusively bound in WT when both TFs were present. As described for other MADS-box TFs [3-5,7,9,20], binding sites were mostly found in promoter regions 5’ of transcriptional start sites, and in smaller proportions in exons, introns and UTRs (Additional file 14). Overall, this distribution was not affected by the presence of FLC (Additional file 14).

For FLC, a total of 315 binding sites were detected in WT by ChIP-seq and these corresponded to 340 target genes, whereas 419 binding sites and 391 target genes were identified in *svp-41* mutant (Figure 2B; Additional file 13). Only 183 genes were detected in both genotypes (Figure 2B), among which were *SEP3* and *SOC1* that were also found in SVP:GFP ChIP-seq. Among the FLC target genes, 157 were detected only in the presence of SVP - again *TEM1* was one of them - but the total number of targets was lower in WT compared with *svp-41*. In this case, the proportion of unique genes did not significantly differ between the two genotypes (*P* = 0.1, Chi-square test). As for SVP, FLC peaks were mainly located in promoter regions in both genotypes (Additional file 14). Several binding sites for each TF were validated by ChIP quantitative PCR in independent experiments (Figure 2C).

The ChIP-seq data indicated that the presence of one of these MADS-box TFs influenced the binding of the other. SVP genome-wide targets approximately doubled in the presence of FLC. On the other hand, FLC binding sites were greatly altered by the presence of SVP, but in a qualitative rather than a quantitative manner, as the total number of targets did not increase. These results indicate that target recognition by complexes of MADS-box TFs can be substantially altered by the expression of their MADS cognate partners.

### Combinatorial binding and joint genomic occupancy of FLC and SVP

To investigate co-regulation of target genes by FLC and SVP their binding sites were compared. First, the sites at which FLC and SVP bound DNA independently of each other were identified. Of the 303 genes recognized by SVP:GFP in *flc-3* background, 87 (29%) were also bound by FLC in *svp-41* (22% of the FLC targets *in svp-41*). Therefore, when FLC and SVP bind independently, about 20 to 30% of their target genes are shared. The presence of these common targets (Figure 2D, top left) and the co-occurrence of binding sites (Figure 2D, bottom) suggests that these TFs can regulate the same pathways independently of each other. Interestingly, among those targets where the proteins bind independently are the flowering integrator genes *FT* and *SOC1*. In agreement with this observation, signalling allocation analysis of *FT* and *SOC1* in the transcriptome data indicates that FLC:SVP is not required for repression of these genes (Additional file 9). On the other hand, 67 genes appear to be bound by both TFs only in the WT context (Figure 2D, top right).

Similarly, common regions bound by SVP:GFP in *flc-3* and in WT (Figure 2A), and also those shared by FLC in *svp-41* and in WT, were very highly correlated (Figure 2E). This analysis suggests that for these common targets the single proteins and the FLC:SVP heterodimer bind to the same regions.

In summary, our results indicate that when FLC and SVP regulate the same target genes in the absence of the other protein they bind to common genomic regions (Figure 2D, bottom). This observation suggests that for certain targets these factors can compensate for loss of activity of the other, acting redundantly and providing robustness to the system. Furthermore, at targets recognised by individual TFs and the complex, they locate to the same binding regions (Figure 2E).

### Peak analysis reveals quantitative and significant differences in SVP and FLC binding sites

In order to better elucidate the basis of the differences that we observed between the ChIP-seq datasets obtained in the different genetic backgrounds (Figure 2A,B), we performed differential binding analyses [50,51] and quantitative comparisons of peak height [52] (Materials and methods; Additional files 12, 15 and 16).

**Figure 3:**
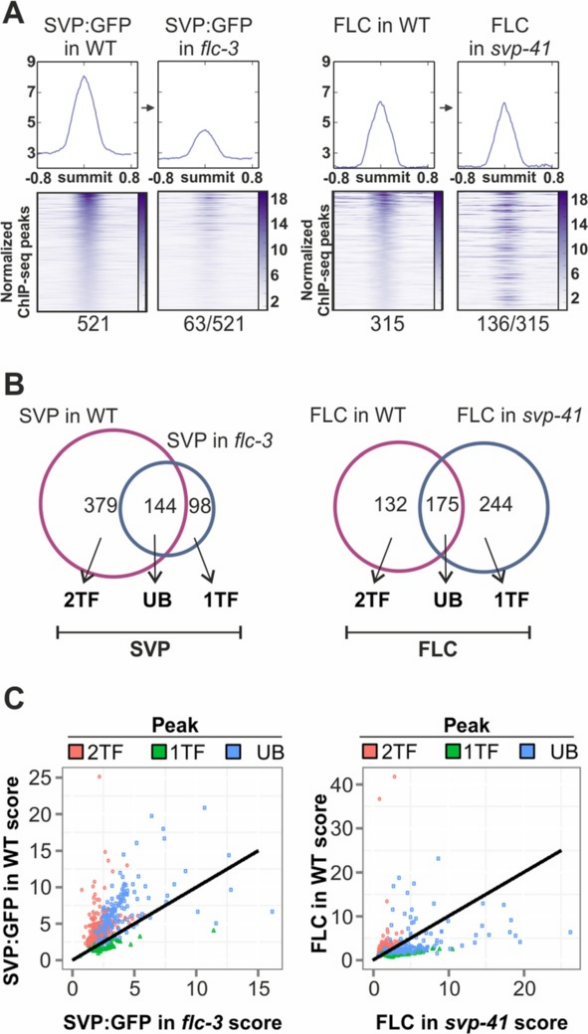
**Normalized fold-change between ChIP-seq peak heights and peak shape analysis by functional PCA techniques show differences in binding behaviour. (A)** Left panels for SVP: heat map of 521 TF binding sites for SVP:GFP in FLC WT for regions ±750 bp around the peak summits (the binding regions were sorted according to their maximum median value), and heat map for SVP:GFP binding intensity in *flc-3* mutant. Regions and ordering are the same in both heat maps. Right panels for FLC: heat map of the 315 TF binding sites for FLC binding in SVP WT for regions of ±750 bp around the peak summits and heat map for FLC binding in *svp-41* mutant. Regions and ordering are the same in both heat maps. Summary images above the heat maps plot the median profiles for each experiment. Values below the heat map indicate significant differentially occurring binding events (adjusted *P* ≤ 0.05) detected by comparison between the paired panels. **(B)** FLC and SVP ChIP-seq datasets used for analysis. Labels: 1TF, peaks enriched exclusively when only one TF is present; 2TF, peaks enriched exclusively when both TFs are present; UB, (ubiquitous) peaks enriched in both conditions. **(C)** Scatter-plot of the normalized peak scores calculated as in Bardet *et al*. [52] for SVP and FLC ChIP-seq peaks. 2TF (379 and 132 peaks, respectively), UB (144 and 175 peaks, respectively) and 1TF (98 and 244 peaks, respectively). No change in fold-enrichment is represented by the black line.

Differential binding in the two genotypes was examined for each TF in a region ±750 bp around the peak summits (Additional file 12). The peaks in each genotype were identified independently and then each peak was compared with the corresponding region in the other genotype. The 521 SVP:GFP peaks in WT were compared with the corresponding regions in *flc-3*, and 63 significant differences were detected (Hotelling’s T^2^ test, adjusted *P*-value ≤ 0.05; Figure 3A; Additional file 16). By contrast, the reciprocal comparison revealed one significant difference in 245 peaks (Additional file 17). However, a similar number of differences was found by comparing FLC peaks in WT with the same regions in *svp-41* (Figure 3A) or FLC peaks in *svp-41* with the corresponding regions in WT (Additional file 18). Median profiles show that, overall, peaks in SVP:GFP in WT are more read-enriched than the same regions observed in the *flc-3* (Figure 3A). Interestingly, the FLC binding pattern is the reverse, showing stronger binding in the *svp-41* mutant genetic background (Additional file 18).

To further explore TF binding strength in our data a quantitative analysis of peak changes was performed, dividing first the consensus lists of regions for FLC and SVP into three groups: peaks present in both WT and the mutant for the interacting TF referred to as ubiquitous (UB); peaks only identified in WT, that is, when both TFs were present (2TF); and peaks only identified in the mutant (1TF) (Figure 3B). Of 144 SVP:GFP UB binding regions, 120 (83%) presented higher binding in the presence of FLC, whereas only 24 presented decreased binding (Figure 3C). By contrast, for FLC, only 47 of 175 UB regions (27%) showed higher binding when SVP was present (Figure 3C). Thus, the presence of FLC quantitatively increased peak height for SVP:GFP whereas FLC showed no strong dependency for binding strength on the presence of SVP. On the other hand, SVP has a more qualitative effect on binding of FLC.

### *Cis*-regulatory elements vary among genotypes

**Figure 4:**
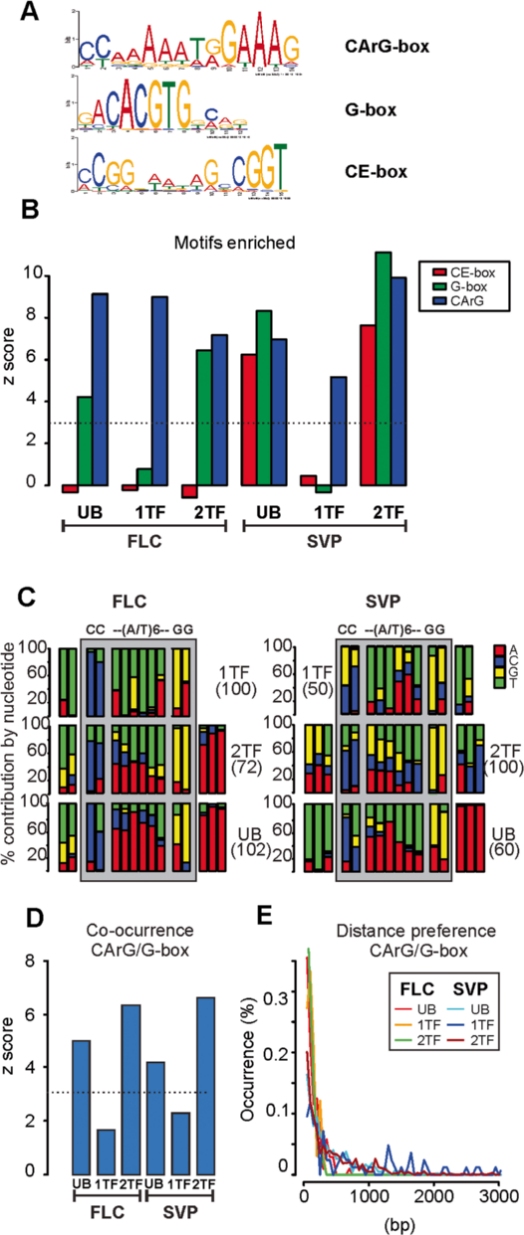
**The FLC:SVP complex binds to distinctive** ***cis*** **-elements. (A)** The sequence logos for CArG-box, G-box and CE-box. **(B)** Bar plot showing motifs enriched in different ChIP-seq peak regions. The CArG-box (blue) was enriched in all ChIP regions whereas the G-box (green) was enriched only when both the SVP and FLC binding site co-localized. The newly identified CE-box motif (red) was enriched only in UB and 2TF subsets of the SVP:GFP ChIP dataset. **(C)** Nucleotide distribution at the *de novo* discovered CArG-box (CC(A/T)_6_GG) motif for FLC and SVP ChIP-seq regions defined in **(B)**. The A/T stretch shows differences in its nucleotide distribution for 1TF, UB and 2TF. The CArG-box motif is highlighted in grey. **(D)** Bar plot showing significant co-occurrences observed for CArG-box and G-box. Significant co-occurrences were observed for CArG-box and G-box when both FLC and SVP binding sites co-localized. **(E)** Histogram displaying the distribution of relative distance observed between nearest CArG-box and G-box occurrences for the different datasets. For FLC binding: 1TF (orange), UB (red), 2TF (green). For SVP binding: 1TF (blue), UB (cyan), 2TF (brown). CArG-box and G-box motifs most often occur within a sequence window of 100 to 200 bp.

To study whether the sets of binding sites of FLC, SVP and FLC:SVP could be explained by the presence of different CArG-boxes or other sequences we performed *de novo* motif discovery using MEME [53]. CArG-boxes bound by most plant MADS-box proteins correspond to the canonical sequence CC(A/T)_6_GG, although variations in the distribution of A and T nucleotides in the A/T stretch or its length have been described [54-56], even for the same TF at different developmental stages [57]. DNA regions bound by FLC and SVP were divided as before into three groups (UB, 1TF, 2TF; Figure 3B). Three different motifs were enriched in the dataset (Figure 4A). The canonical CC(A/T)_6_GG motif was found to be the most enriched in all genotypes, whether FLC or SVP and 1TF, 2TF or UB data were used (Figure 4A,B) and CArG-boxes with different A/T stretches (for example, 5 and 7 bp length) were also enriched in the three categories (UB, 1TF, 2 TF) (Additional file 19). Therefore, FLC:SVP did not appear to recognise CArG-boxes with different A/T stretch lengths than those bound by the TFs acting independently. Nevertheless, analysis of the position weight matrix revealed differences between the recognition sites for the heterodimer and for the TFs acting independently (Figure 4C). We identified differences in A/T distribution. For 1TF the A/T stretch clearly favours the T nucleotide for most of the positions, whereas for 2TF or UB this preference is less pronounced and in fact inclined more towards the A base (Figure 4C; Additional file 20). This difference likely contributes to sequence-specific recognition capabilities for the heterodimer and TFs acting independently. *De novo* motif discovery also detected other motifs significantly enriched in the peak sequences. G-box motif (CACGTG) was found in UB or 2TF peaks but not in the 1TF set (Figure 4A,B; Additional file 21). The selective enrichment of the G-box suggests that basic helix-loop-helix (bHLH) or bZIP TFs might associate more often with the FLC:SVP heterodimer than with complexes containing only one of the factors. Significant co-occurrence of CArG and G-box motifs was observed when FLC and SVP were both present (Figure 4D) and this appears to occur within a distance interval of 100 to 200 bp (Figure 4E), indicating that both motifs occur in the same binding region. G-boxes were previously found adjacent to SVP binding sites [9]. In addition, a previously undescribed Complex-Enriched motif (CE-box, sequence [CA]CGG[GT][AT]A[TG][AT]GCCGGT) was enriched only among the targets in the 2TF and UB of SVP ChIP-seq dataset (Figure 4A,B; Additional file 21).

In summary, the data suggest that the heterodimer binds preferentially to different CArG sequences than the individual TFs, and might specifically interact with bHLH or bZIP TFs at G-boxes enriched adjacent to the CArG-boxes. Also, a novel motif (CE-box) was detected that is exclusively present adjacent to sites at which SVP binds in genotypes where it can form a complex with FLC.

### Targets of the complex and processes governed by FLC and SVP in *Arabidopsis* flowering

To gain further insight into the biological roles of FLC and SVP acting as a complex or individually, the genes targeted by these TFs in WT and mutant backgrounds were analysed. First, genes bound in WT but not in mutant genotypes were selected. The genes present in either 2TF group were bound by FLC or SVP only in WT, and therefore were combined in one set that contained 643 genes. This set was referred to as complex-enriched genes, because it was expected to contain all the genes bound by the complex, but might also contain some genes bound in WT by only one of the TFs. A more stringently selected set of genes bound by the complex is represented by those present in both 2TF groups. This set contained 67 genes that after stringent individual inspection was reduced to 45, and these were referred to as complex-exclusive genes.

**Figure 5:**
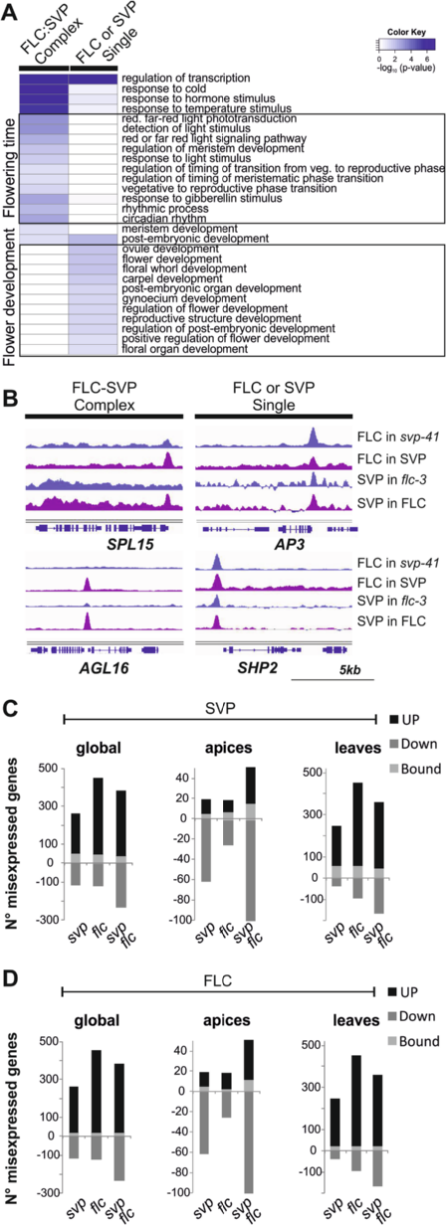
**Integration of transcriptional profiling and binding site data. (A)** GO term enrichment analysis of high-confidence targets that were found only when both TFs are present (labelled FLC:SVP complex) or that were independent of complex formation (labelled FLC or SVP single). **(B)** Local enrichment of SVP:GFP and FLC binding in four different regulatory regions. *AP3* and *SHP2* are complex-independent, while *SPL15* and *AGL16* are bound in a complex-dependent manner. Bar represents a 5 kb window. **(C,D)** Proportion of direct targets of SVP:GFP **(C)** and FLC **(D)** among genes identified as up- or down-regulated in the *svp-41*, *flc-3* and *svp-41 flc-3* in the whole set (left) or divided between apices (middle) and leaves tissue samples (right).

These gene lists were analysed according to their functional annotation [58] and Gene Ontology (GO) terms enriched in the different datasets were compared. In all datasets, as expected, overrepresentation of genes related to regulation of transcription was detected, indicating that FLC and SVP have indirect as well as direct effects on transcriptional regulation, expanding their impact to other biological processes. TFs present in the complex-enriched set or in those sets bound by the TFs acting independently consist mainly of proteins with MADS-box, AP2 or bHLH domains. Interestingly, considerable differences between enriched GO categories in the complex-enriched and individual factors gene sets were detected (Figure 5A). A high enrichment in terms related to flower development was observed in the datasets obtained when the proteins were acting independently from each other (Figure 5A). These genes include several encoding other MADS-box TFs such as *SEP3*, *SHATTERPROOF 2* (*SHP2/AGL5*), *APETALA3* (*AP3*), *SOC1* and *SVP* (Figure 5A,B; Additional file 22). In contrast, genes involved in flower development are not as prominent among complex-enriched targets, but rather these include many genes controlling flowering time and environmental responses (Figure 5A,B). The GO analysis also highlighted the enhanced function of the complex in responses to cold and temperature stimulus (Figure 5A). Regulation of flowering by the complex appears to be achieved via diverse pathways such as photoperiod or circadian clock and the GA pathway (see later). *AGAMOUS LIKE-16* (*AGL16*) encodes a MADS-box TF that was described as a target of SVP and FLC in previous studies [3,4]. Our data indicate that its binding is dependent on the FLC:SVP complex (Figure 5B), and it falls both in the complex-enriched and complex-exclusive lists. Its expression pattern correlates with this finding, being increased similarly in each single mutant and in the double mutant compared to WT (Additional file 22). Interestingly, AGL16 was recently shown to be involved in flowering-time control and to interact with FLC [59]. The ChIP-seq normalized read alignments for the 30 regions (45 associated genes) fulfilling the requirements for complex-exclusive binding are shown in Additional file 25 along with descriptions of all the genes present. Twenty-four of these 45 genes were previously described as targets of SVP or FLC [3,4,9].

Taken together, our functional analyses suggest that individually FLC and SVP, probably coupled with other TFs, predominantly control flower development, whereas the FLC:SVP heterodimer acts as a major regulator of flowering time.

### Integration of transcriptional profiling and binding site data

The correlation between DEGs and direct targets obtained by ChIP-seq was then assessed to determine how many of the transcriptional changes are due to direct binding of FLC and SVP. The DEGs in leaves and apices were combined and compared with the list of target genes obtained from seedlings in the ChIP-seq experiments. This global analysis showed that 15 to 20% of DEGs in all the mutants were direct targets of FLC or SVP (Figure 5C,D, left; Additional file 23). Furthermore, 90% of the direct targets that were differentially expressed showed increased expression upon loss of the regulatory input of the TF. To uncover tissue specificity, DEGs in apices and leaves were compared with direct targets of FLC and SVP. Of the up-regulated genes in leaves and apices, 20% were bound by at least one of the TFs (Figure 5C,D). Although in apices the majority of the differential transcripts were down-regulated in the mutants (Figure 1B), less than 2% were direct targets of FLC or SVP. Therefore, a high proportion of the down-regulated genes in apices of mutants are indirectly regulated by FLC or SVP. These data support the idea that FLC and SVP act predominately as repressors of transcription.

### Regulation of gibberellin-related genes partially depends on the FLC:SVP complex

**Figure 6:**
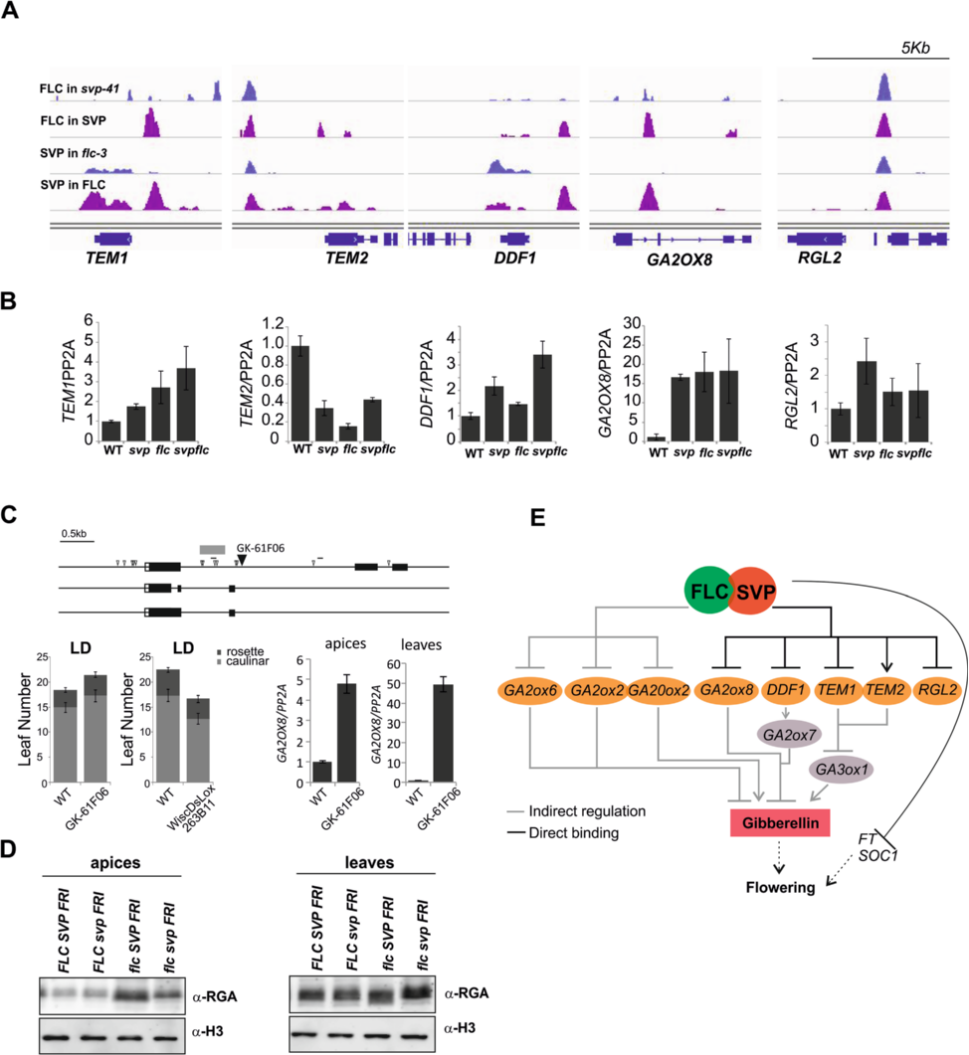
**Coordinated regulation of GA-related genes by FLC and SVP. (A)** Local enrichment of SVP:GFP and FLC proteins bound to genes involved in GA signalling: *TEM1*, *TEM2*, *DDF1*, *GA2OX8* and *RGL2* (as inferred by ChIP-seq). Bar denotes a 5 kb window. **(B)** Transcript levels of *TEM1*, *TEM2*, *DDF1*, *GA2OX8* and *RGL2* in apices of different mutants. Expression was measured by qRT-PCR. **(C)** Characterization of *ga2ox8* mutant alleles. Top: gene model of *GA2OX8*. The GK-61 F06 insertion site is depicted by a filled triangle. Empty triangles denote CArG-boxes. The grey box indicates the FLC:SVP binding site identified by ChIP-seq. Bottom left: number of leaves at flowering time for WT and two alleles of *ga2ox8* (GK-61 F06 and WiscDsLox263B11) measured under LD photoperiod. Bottom right: transcript levels of *GA2OX8* in leaves and apices determined in 2-week-old plants by qRT-PCR. **(D)** Western blot analysis detecting RGA protein levels in apices and leaves of WT and single and double mutants grown for 2 weeks under SDs. **(E)** Model for FLC and SVP regulating the GA signalling pathway. Direct and indirect regulation described in this study are illustrated as shown. Mean values are accompanied by standard deviation in **B** and **C**.

Several genes related to GA biosynthesis and response were present among the high-confidence FLC and SVP direct targets as well as DEGs (Additional file 23) and were also found to be overrepresented in the GO terms analysis (Figure 5A; Additional file 24). Moreover, SVP was recently shown to indirectly reduce transcription of a GA biosynthetic enzyme to lower GA biosynthesis and delay the floral transition [32]. Therefore, whether regulation of genes involved in GA-related processes depends on the FLC:SVP complex was examined. *TEMPRANILLO* (*TEM*) genes encode TFs that delay flowering by directly repressing *FT* and reducing GA levels [60,61]*.* TEM1 directly binds to and represses transcription of paralogues encoding GIBBERELLIN 3-OXIDASE, *GA3OX1* and *GA3OX2*, an enzyme required for biosynthesis of bioactive GAs [61]. *TEM1* and *TEM2* were among the direct targets of FLC and SVP and *TEM1* was in the complex-exclusive set (Figure 6A). *TEM1* and *TEM2* mRNA levels were also changed in leaves and apices of single and double mutants, suggesting that binding of FLC and SVP regulates their expression (Figure 6B). Interestingly, in agreement with what was found for FLM and SVP [8,9], *TEM2* expression decreased up to three-fold in the *svp flc* double mutant, suggesting that it might be activated by FLC and SVP binding (Figure 6B). By contrast, for *TEM1* the FLC and SVP proteins act as repressors (Figure 6B). In addition, *GA3OX1* mRNA levels were increased in the mutant (Additional file 23), probably due to an indirect effect of the misregulation of the *TEM* genes as it was not detected as a direct target of either FLC or SVP.

*DWARF AND DELAYED FLOWERING 1* (*DDF1*) encodes an AP2-type TF that promotes expression of the GA catabolic enzyme *GIBBERELLIN 2-OXIDASE 7* (GA2OX7) [62]. *DDF1* was associated with a complex-exclusive peak (Figure 6A; Additional files 25 and 26) and *DDF1* mRNA levels were two-fold higher in single mutants of either *svp-41* or *flc-3* than WT and up to four-fold higher in the double mutant (Figure 6B). Therefore, FLC and SVP additively repress expression of this activator of GA catabolism through direct binding to its promoter. Furthermore, *GIBBERELLIN 2-OXIDASE 8* (*GA2OX8*), a homologue of *GA2OX7*, was also present in the complex-exclusive list (Figure 6A; Additional file 25). Regulation of this gene was further examined because ectopic expression of *GA2OX8* was shown to delay flowering in *Arabidopsis* [63]. The complex-exclusive binding was validated independently by showing that FLC and SVP bind to canonical CArG-boxes of the first intron in *GA2OX8* (Figure 6A; Additional files 26 and 27). FLC and SVP repress *GA2OX8* expression in a complex-dependent manner as judged by the transcription profile of the respective single and double mutants (Figure 6B). To functionally analyse the significance of this site, a T-DNA insertion at the intronic region, GK-61 F06, was analysed (Figure 6C; Additional file 27). Strikingly, the mutant flowered later than WT under LDs and SDs (Figure 6C; Additional file 27), a phenotype associated with overexpression of *GA2OX8* [63]. In agreement with the late-flowering phenotype, the *GA2OX8* transcript in GK-61 F06 was more abundant than in WT (Figure 6C), indicating that the insertion causes overexpression of the gene in leaves and apices. Disruption of the intron to which FLC and SVP bind therefore appears to cause up-regulation of *GA2OX8* mRNA. By contrast, an independent mutant allele of *GA2OX8* (WiscDsLox263B11) carrying a T-DNA insertion in the first exon (Additional file 27) caused early flowering (Figure 6C; Additional file 27). Lastly, other direct targets of FLC and SVP include components associated with GA signalling. *RGA-LIKE2*, which encodes a DELLA protein repressor of GA signalling that is rapidly degraded in response to GA [64], is also bound by FLC and SVP (Figure 6A; Additional file 26). Here, no significant misregulation of *RGL2* mRNA was detected in leaf or apical RNA samples of the mutants (Figure 6B). Finally, the levels of the RGA protein DELLA fluctuate in single and double *svp* and *flc* mutants (Figure 6D), consistent with effects on GA abundance and signalling in these genotypes.

Previously, higher levels of bioactive GA were detected in the *svp* mutant due to deregulation of *GA20OX2* expression, which is indirectly repressed by SVP [32], and FLC was reported to bind to one gene encoding a GA biosynthetic gene and to GID1c, a member of the GA receptor family [3]. Here we show that SVP and FLC co-operatively regulate several genes with effects on GA biosynthesis, catabolism and signalling (Figure 6E), suggesting that modulating the effects of GA constitutes a major function for these TFs.

## Discussion

The *Arabidopsis* MADS-box TFs FLC and SVP interact to form a complex and each represses the transition from vegetative growth to flowering. Here we studied their functional interdependency at the genome-wide level. Transcript profiling and ChIP-seq studies were performed in WT plants in which both TFs are active as well as in mutants lacking activity of one or both TFs. The landscape of binding sites and regulated genes differed dramatically when both factors were active compared with when they functioned individually. Strikingly, a subset of binding sites that is recognized by each factor only when the other is present identified genes that are regulated specifically by the FLC:SVP complex. Genes involved in flowering time or environmental responses were enriched among those recognized by the complex whereas genes with developmental functions during flower development were over represented among those recognized by the TFs acting individually. Our study demonstrates at the genome-wide level how the functions of these plant MADS-box TFs with pivotal roles in reproductive development are influenced by their cognate partners.

### Genetic interactions of SVP and FLC on flowering time and gene expression

SVP and FLC contribute to several endogenous and environmental pathways that govern floral induction in *A. thaliana* [1]. Their major function is to delay or prevent flowering in non-inductive environments so increasing the amplitude of the flowering response to inductive cues. Genetic analysis demonstrated that these two TFs have overlapping functions [12,33]. SVP FLC plants are strongly delayed in flowering under inductive LDs or non-inductive SDs, although they can be accelerated to flower by exposure to vernalization that reduces *FLC* transcription. Mutation of *FLC* or *SVP* is sufficient to cause much earlier flowering under inductive LDs, while double mutants exhibit extreme early flowering even under non-inductive SDs. The genetic data demonstrate that although these TFs interact to form a complex, each protein must retain a function in the absence of the other. MADS-box TFs interact with other TFs of the same class to form higher order complexes [24], so individual functions of SVP and FLC probably depend on interactions with other members of the family. Indeed, SVP interacts with a broad class of MADS-box TFs, including AP1, CAL, SEP3, AGL16 and FLM [8,11,35,59,65], whereas FLC interacts with at least MAF3, MAF4 and FLM [36].

To define the contribution of FLC or SVP acting individually, genome-wide transcriptome analysis of the WT and single and double mutants in apices and leaves was carried out. Both genes are expressed in these tissues and influence flowering when expressed in either region [31,32]. As expected from the related mutant phenotypes, a considerable overlap in the function of FLC and SVP was observed at the transcriptome level, although each TF also showed specific effects. FLC controlled twice as many genes in leaves as SVP, and mutations in *FLC* have an effect on vegetative phase change reflected in leaf heteroblasty [44], whereas this has not been described for *svp* mutants. However, mutations in *SVP* do increase GA levels leading to changes in chlorophyll accumulation in leaves and to leaf petiole elongation [32]. In contrast to leaves, SVP had a more significant effect on gene expression in the apex than FLC. The lower number of genes misexpressed in apices of *flc* mutants nevertheless included flowering-time genes such as *SOC1*. SVP has defined functions in flower development in part by interacting with other MADS proteins involved in this process [11,65] and such interactions might contribute to a broader role in the apex.

Interestingly, a set of leaf and apex genes that are bound specifically by the FLC:SVP complex in seedlings was identified (Figures 2 and 5; Additional file 25). Expression levels of a subset of these genes showed similarly increased levels of expression in the single and double mutants, demonstrating that both proteins are required for their repression. Similar comparative transcriptome data were reported for PISTILLATA and APETALA3 MADS-box factors that interact to specify petal development [10]. In that case similarities in the transcriptome were emphasized but the single mutants were not compared directly with the double mutant [10]. The flexibility we observed for FLC and SVP is likely a feature of the ability of these TFs to interact with additional MADS-box proteins to substitute for the loss of one partner or to provide distinct specificities. Both FLC and SVP interact with other MADS-box TFs, as described above. Conceivably each of these TF complexes might bind to specific sets of targets as we show here for groups of genes that are recognized uniquely by the FLC:SVP complex but not by either protein acting individually.

### Effect of interactions between SVP and FLC on target site occupancy

Genome-wide identification of binding sites for FLC and SVP revealed that 15 to 25% of the transcript changes were caused by direct regulation and that SVP and FLC act almost exclusively as repressors (Figure 5). Although the ChIP was performed on seedlings and the RNA expression analysis on apices or leaves, the proportion of misexpressed genes directly bound by the TFs is in a similar range to what has been observed previously for other MADS-box proteins [7]. Inducible forms of TFs or induction of artificial microRNAs that lower TF activity at specific times can be used to reduce the indirect effects of TFs and increase the proportion of direct targets identified in transcript profiling experiments [7,10].

Genome-wide targets of FLC and SVP were previously described. Studies of SVP identified 2,982 targets by ChIP-seq in SD-grown plants [4] while Tao *et al*. [9] reported 328 genes associated with SVP using ChIP to chip on plants grown under LDs. In WT, we identified 773 genes associated with SVP binding, which included 71 genes in common with Tao *et al*. [9], and 54 with Gregis *et al*. [4] (Additional file 28). These differences might be caused by environment, the genotype (in one case overexpressing plants were used [9] while the other utilized the endogenous promoter [4]), the technology used to identify ChIP enriched fragments (ChIP-chip, ChIP-seq using a GAII sequencer or a Hi-Seq 2000 in our study) and the bioinformatics approach employed to analyse the data (replicate treatment, FDR versus IDR [50] and alignment tools). Nevertheless, our data were validated in triplicate biological replicates and using recently developed statistical tools, providing additional support for these binding sites. Strikingly, our SVP data showed higher overlap with published FLC target lists identified by ChIP-seq, 141 genes in common with Deng *et al*. [3] and 223 genes in common with our FLC data. Overlap with FLC datasets from the other SVP studies [4,9] was less than 60 genes in all cases. The comparison of FLC targets identified by Deng *et al*. [3] and by us showed a 40% overlap (Additional file 28). This high overlap found under different environments and performed independently in different laboratories provides strong support for our SVP and FLC target lists.

Variation among sites recognised by the TFs acting individually or together was found at the level of *cis*-elements (Figure 4). Canonical CArG boxes appear equally in genes bound by the single TFs or by the complex, but other *cis*-elements, such as G-boxes, were encountered more frequently at sites where the complex is formed. The presence of the G-box neighbouring the CArG boxes (Figure 4D) could be part of a mechanism by which the complex and single factors discriminate between targets. For example, the FLC:SVP complex might associate with a bHLH or bZIP TF that recognizes G-boxes to control transcription of those target genes. This is in part supported by the transcriptome analysis as signal allocation analyses identified a group of genes whose expression pattern might be explained by the presence of a third factor (Additional file 6). Such a mechanism might strengthen the affinity for weak CArG boxes. Interestingly, a new motif was also found only in genes bound by SVP complexes (Figure 4).

The functional annotation analysis suggests that those genes regulated only by the complex have distinct biological functions to those regulated by the factors acting individually. Many genes involved in flower development were bound by FLC or SVP independently of the presence of the other TF. Several other MADS-box proteins that interact with FLC and SVP are involved in flower development, including SEP3, SHP2, PI, AP3 and SOC1. Therefore, interaction of these with either FLC or SVP could contribute to flower development and reduce the requirement for the complex. By contrast, the importance of the complex in controlling floral transition was evident because genes involved in several flowering pathways were overrepresented among its targets. The complex-enriched set contained the floral-induction related genes *TEM1*, *TEM2*, *AP2*, *PIF3*, *TOE*s, *miR172*, *CONSTANS-LIKE* genes, *SPL15*, *GA2OX8* and *DDF1* among others. The integrator genes *SOC1* and *FT*, whose regulation by FLC and SVP strongly contributes to flowering-time repression [12,25,31,32], were bound by FLC and SVP acting independently. However, their effect is likely enhanced by regulation of other flowering-time genes by the complex, which could fine-tune the flowering response by influencing a wider range of pathways. Several of these pathways ultimately converge on *FT* so that FLC and SVP regulation of *FT* transcription could be achieved both by direct binding and indirectly through the complex regulating upstream pathways. For example, *TEM1/TEM2*, to which the FLC:SVP complex binds, encode direct repressors of *FT* transcription [60]. Overall we found that the FLC:SVP complex regulates multiple pathways within the flowering network (Figure 6E).

In flowering plants, multiple MADS-box TFs share similar structures and form complexes of dimers or tetramers. Therefore, they are able to multimerize in a combinatorial fashion, although the ultimate composition of these complexes *in vivo* is only beginning to be deciphered [66]. We elucidated mutual interdependencies between the floral repressors FLC and SVP by performing ChIP-seq independently for each TF in different genetic backgrounds. This method allowed us to indirectly assess which genes are regulated by the complex. Future development of methods to detect the complex directly bound to DNA will further clarify which genes are regulated by FLC and SVP acting together.

### Regulation of gibberellin biosynthesis and signalling by FLC SVP

The targets of FLC and SVP included several genes implicated in GA biosynthesis or signalling. GA has long been known to promote flowering of *Arabidopsis*, most strongly under SDs [67] but also under LDs [68-71]. However, how GA biosynthesis and response are regulated by and intersect with other flowering pathways that are comprised mainly of TFs is still poorly understood. SVP was recently shown to indirectly reduce *GA20OX2* mRNA abundance and thereby GA levels. Also, increased *GA20OX2* mRNA and GA levels contribute to the early flowering of *svp* mutants [32]. Furthermore, *GA3*, which encodes an ent-kaurene oxidase that catalyzes early steps in GA biosynthesis, was identified as a target of FLC, as was GID1C, which is a member of a small gene family encoding GA receptor proteins [3]. However no effect of FLC on the expression of these genes was described. By contrast the TEM1 and TEM2 TFs that delay flowering are repressors of genes encoding the GA biosynthetic enzyme GA3ox [61].

Our data indicate that FLC and SVP have several roles in regulating GA metabolism, but that these appear to have opposing effects (Figure 6E). For example, a major direct effect of FLC:SVP appears to be to repress expression of GA2 oxidases, which are GA catabolic enzymes. FLC:SVP bind directly to and repress the genes encoding *GA2OX8* and DDF1, a transcriptional activator of *GA2OX7* [62]. In addition, *GA2OX6* and *GA2OX2* were not bound by FLC:SVP but were identified as upregulated DEGs in the mutants. The complex, therefore, represses the expression of enzymes required to reduce GA levels, and the transcripts encoding these enzymes are increased in abundance in early flowering *flc svp* double mutants. Paradoxically, increased expression of these enzymes in transgenic plants is associated with late flowering and reduced GA levels [63], whereas *flc svp* mutants are early flowering and *svp* mutants contain elevated GA levels [32]. Genes encoding GA2 oxidases often exhibit precise spatial and temporal expression patterns [72,73], and the FLC:SVP complex might regulate these genes in different cells to those in which GA levels must rise to promote flowering. In addition, FLC:SVP negatively regulates *TEM1* and positively regulates *TEM2*, which encode repressors of *GA3ox1*, whose product is an enzyme required for GA biosynthesis. In agreement with this binding, *flc svp* double mutants express higher levels of *TEM1* mRNA and lower levels of *TEM2* in leaves and apices as well as higher levels of *GA3ox1* mRNA in leaves. *TEM1* and *TEM2* might be expressed in different cells or at distinct times so that the effect of both genes confers a spatial pattern of GA biosynthesis that could be important in modulation of the floral transition and related processes. *TEM1* and *TEM2* were also reported to be bound by SVP and SOC1 [9]. In total seedling RNA, *soc1* mutants showed increased *TEM1* and *TEM2* mRNA levels. By contrast, no effect of *svp* mutations was detected, although *SVP* overexpressors caused an increase in *TEM1* and *TEM2* mRNAs [9]. Since FLC:SVP also represses *SOC1*, some of the effects on *TEM1* mRNA detected in *svp flc* mutants might be indirect due to increased *SOC1* expression. Finally, the mRNA encoding the biosynthetic enzyme *GA20OX2* was present at higher levels in single and double mutants, consistent with a previous report [32] and with the early flowering as well as increased GA levels detected for *svp* mutants.

Thus, FLC and SVP have a broad role in regulating GA metabolism and an unexpectedly strong, direct effect in repressing GA catabolism, but integration of this into their role in controlling flowering awaits a more extensive understanding of the spatial and temporal expression of GA metabolism and its regulation during floral transition.

### FLC and SVP as an evolutionarily flexible system

Plant MADS-box proteins were previously described as showing degeneracy, whereby different members can perform similar functions in one condition but distinct functions in another [74]. This feature is believed to support robustness within biological processes while also providing flexibility for adaptation and evolution of the system [75,76]. Our genome-wide analysis of the MADS-box proteins FLC and SVP demonstrates that they function as degenerate elements, which are redundant in some scenarios but have distinct functions in others, and this might facilitate variation in their activity among *Arabidopsis* accessions. Such plants vary tremendously in the contributions of FLC and SVP to flowering control. Winter-annual accessions contain an active, highly expressed *FLC* gene whereas summer annuals do not and this difference confers variation in requirement for vernalization to flower [16,18]. Similarly, some early-flowering Asian accessions show reduced SVP activity [77]. Furthermore, the important role of FLC in vernalization requirement seems to have evolved relatively recently at the origin of the Brassicaceae; although *FLC* orthologues were found in other families by utilizing conservation of synteny, their contribution to vernalization requirement remains unclear [78]. Our data show that in the presence of FLC, the number of SVP binding sites and the strength of their recognition increases (Figures 2 and 3). Therefore, FLC might have evolved such an important role in flowering in the Brassicaceae at least in part by enhancing the activity of SVP. Although FLC clearly also functions independently of SVP. Such overlapping or degenerate functions of these TFs might be important in ensuring that loss of activity of one of them does not impair essential functions and that, therefore, early flowering summer annual accessions can compete in natural populations.

## Conclusions

Our work has general significance in understanding the combinatorial activity of MADS-box TFs. We decoded the genome-wide interdependency of FLC and SVP, two TFs that have crucial roles in flowering control. Our findings show that their activities change when they act individually or as a complex. This behaviour confers flexibility as well as robustness to the regulatory network they govern. Also, their overlapping activities may allow plants in natural populations to tolerate genetic variation at these two genes, and thereby contribute to phenotypic differences in seasonal flowering behaviour observed in nature.

## Materials and methods

### Growth conditions, plant materials and phenotypic analysis

The WT *Arabidopsis* plants used in most of this study represented the *FRI* introgression line (Col FRI) [79]. The deficient WT Col/fri was used in a minor number of experiments (indicated in the legends). Plants were grown on soil under controlled conditions either under LDs (16 h light/8 h dark) or SDs (8 h light/16 h dark) at 20°C. The level of photosynthetic active radiation was 150 μmol m^-2^ s^-1^ under both conditions. The mutant lines described here are: *svp-41* [15], *flc-3* [16], *svp-41 flc-3*, *din10* (SAIL-54-G03), and *ga2ox8* (GK-617 F06, WiscDsLox263B11). *svp-41*, *flc-3* and *svp-41flc-3* were crossed with Col FRI-Sf2 to obtain an active FRI background. Plants used for ChIP experiments are described below in the 'ChIP experiments' section. For flowering phenotype determination, the numbers of rosette and cauline leaves were counted at flowering for at least 12 individual plants.

### Microarray expression analysis

Seedlings from *SVP FLC FRI*, *SVP flc-3 FRI*, *svp-41 FLC FRI* and *svp-41 flc-3 FRI* genotypes were grown for 2 weeks under SD conditions or 2 weeks under SDs and then transferred for 2 days under LDs. Leaf and apex tissue was harvested at Zeitgeber 8 (ZT8). Total RNA from three biological replicates was isolated with an RNAeasy extraction kit (Qiagen, Hilden, Germany) and probed to an AGRONOMICS1 Tiling array [80]. Probe signal values were subjected to the robust multi-array average (RMA) summarization algorithm [81] using the ‘rma’ function in the R environment. The following models were fit to log_2_ expression values using the ‘lmFit’ function in the limma package in R: S_gytr_ = GYT_gyt_ + R_r_ + ε_gytr_, where S is log_2_ expression value, GYT is genotype:condition:tissue interaction, and R and ε are the random factors: R is biological replicate and ε is the residual. The ‘ebayes’ function in the limma package was used for variance shrinkage in calculation of the *P*-values and the Storey’s q-values were calculated from the *P*-values using the ‘qvalue’ function in the qvalue package. Heat maps were generated by CLUSTER3.0 [82] using uncentred Pearson correlation and complete linkage, and visualized by TREEVIEW [83]. The signalling allocation analysis was performed as previously described in [40,41].

### RNA extraction and quantitative real-time PCR

Total RNA was isolated from leaf or apex tissues using an RNAeasy extraction kit (Qiagen) and treated with RNAse-free DNase (Ambion, Darmstadt, Germany) to remove residual genomic DNA. Total RNA (1 μg) was used for reverse transcription (Superscript II, Invitrogen, Darmstadt, Germany). Transcript levels were quantified by quantitative PCR in a LightCycler 480 instrument (Roche, Basel, Switzerland) using *PP2A* (At1g13320) and *UBC21* (At5g25760) as house-keeping genes. The sequences of the primers used to quantify the expression are listed in Additional file 12.

### ChIP experiments

For ChIP experiments plants were grown under SDs for 2 weeks and above-ground tissue was collected at ZT8. Three biological replicates were performed for all the ChIP assays. For ChIP on SVP we used the line expressing SVP fused to GFP under its own promoter in the *svp-41* mutant, *SVP::SVP:GFP svp-41* [11], and crossed it with flc-3 FRI to obtain *SVP::SVP:GFP svp-41 flc-3 FRI* and *SVP::SVP:GFP svp-41 FLC FRI*. Both lines were compared with the control line, in our case *35S::GFP*. Polyclonal antibody (5 μl) against GFP from Abcam (Ab290) was used to immunoprecipitate chromatin (Abcam, Cambridge, England). For ChIPs on FLC we used the genotypes Col FRI and *svp-41* FRI and these were compared to the control *svp-41 flc-3* FRI. In this case, 2 μl of FLC antiserum was used (kindly provided by Chris Helliway) [3]. After crosslinking the tissue, the ChIP was performed as in Gendrel *et al*. [84] with minor changes. Before proteinase K treatment, samples were treated with RNAse for 1 h at 37°C, purified with MinElute Reaction Cleanup kit (Qiagen) and eluted in 15 μl. ChIP samples were tested for enrichment by quantitative PCR measuring enrichment on the promoter region of *SEP3* [3] and a negative primer on the exon using primers described in Additional file 12. For ChIP-seq, 10 μl from the eluted chromatin was then used for library preparation using Ovation Ultralow DR Multiplex System (NuGen, Leek, The Netherlands). Libraries were sequenced at the Max-Planck-Genome centre Cologne using an Illumina Hi-Seq2500 instrument.

### ChIP-seq data analysis

We followed recommended guidelines in the analysis of ChIP-seq data for quality control, read mapping, normalization, peak-calling, assessment of reproducibility among biological replicates, and post-processing of peaks [50]. Low-quality reads in the raw data (FASTQ files) were filtered out using Parallel-QC 1.0 [85]. Reads kept were then mapped to the *A. thaliana* genome (TAIR10) using Bowtie [86] version 2.0.2 under default parameters. Technical replicates showed very high similarity and were combined. Reproducibility of reads mapped and peaks called was assessed between biological replicates. Peak calling was done using MACS version 2 [87] with a *P*-value cutoff of 1e-3, followed by IDR analysis [88]. Duplicated reads were not considered during peak calling in order to achieve a better specificity [50]. Peaks across the three replicates with an IDR ≤ 0.05 were retained. We further post-processed the peaks using shape analysis implemented in the Bioconductor package NarrowPeaks [51]. Only MACS peaks also detected by NarrowPeaks were considered for further processing. Final sets of peaks were annotated to TAIR10 using the Bioconductor package CSAR [89]. Analysis of peak distribution over exon, intron, enhancer, proximal promoter, 5’ UTR and 3’ UTR was done in R using ChIPpeakAnno [90]. Proximal promoter and immediate downstream were considered, respectively, 3 kb upstream of the transcription start site and 1 kb downstream the transcription end site. Quantitative comparison of peaks was done following the procedure described in [52], without quantile normalization of the peak scores. Differential binding analysis was performed using functional principal component scores (obtained from shape analysis in NarrowPeaks) over a list of aggregated regions across conditions. Genomic regions were declared as significantly different if *P* ≤ 0.05 (Hotelling’s T^2^ test). *P*-values were adjusted for multiple comparison using Benjamini-Hochberg correction [91]. Genes that present significantly different binding profiles for FLC and SVP in, respectively, *svp-41* and *flc-3* mutants as opposed to the WT were tentatively classified as complex-dependent. The plots and heat maps in Figures 2D,E and 3A and Additional files 17 and 18 were generated using deepTools-1.5.3 [92]. Regions were sorted in descendent order by the maximum of the non-overlapping median bin calculated over the regions’ length. Missing data are indicated as zero. Any region containing an intensity value >20 was skipped. ChIP-seq data visualization in Figures 5B, 6A and Additional files 22 and 23 was done using IGV [93]. Detailed description of the analysis can be found in Additional file 12.

### Gene ontology analysis

Significant GO terms were identified using the Functional Annotation Tool from DAVID Bioinformatics resources [58,94].

### Motif analysis

For each ChIP experiment we first generated three subsets of peaks (and associated sequences): peak regions in (the experiments with) both genotypes (UB), peaks present only for the WT genotype (2TF), and peaks present only for the respective mutant genotype (1TF). ChIP regions for all six scenarios (UB, 2TF, 1TF for SVP and FLC) were screened for enriched *cis*-elements. We used MEME [53] with ‘zoops’ (zero or one per sequence) model and then ‘anr’ (any number of repeats) for *de novo* motif identification. MEME parameters were set to find the 10 most significant motifs with length between 5 and 20 nucleotides. TOMTOM [95] (from MEME suite) with default parameters (+ matching all motifs) was used to match identified motifs with the JASPAR CORE motifs database.

### CArG-box searches with different spacer lengths

To assess the frequency of CArG-boxes with different spacer lengths, we generated position weight matrices of CArG variants with different spacer lengths ranging from 5 to 7 nucleotides. Subsequently, MOODS [96], a position weight matrix search tool, was used to re-screen ChIP regions for CArG-box variants. Only matches with *P*-value <0.001 were considered significant.

### Permutation test for *de novo* identified *cis*-elements

To confirm enrichment of *de novo* identified *cis*-elements 1,000 sets of regions with similar length to ChIP regions were selected randomly from the *A. thaliana* reference sequence (TAIR10). MOODS was used to re-screen ChIP and random regions. Average numbers of sequences with CArG-box and G-box as well as standard deviation in random sets were calculated to generate Z-scores for each motif for each of the six scenarios. Z-scores above 3 were considered as significant.

### Distance preference for CArG-box and G-box

To check whether CArG-boxes and G-boxes preferentially occur within a specific distance range in any of the six scenarios, we searched for all occurrences of CArG-boxes and G-boxes and calculated the distances between nearest *cis*-elements of both categories.

### Immunoblots

Plant protein extracts of 2-week-old SD grown plants dissected for apices and leaves at ZT8 were prepared in extraction buffer (50 mM Tris-HCl, 150 mM NaCl, 0.5% Triton-X100, 10 μM MG132, 0.1 μM PMSF and Protease Inhibitor Cocktail, Sigma-Aldrich (Sigma-Aldrich, St. Louis, MO, USA) and a total of 30 μg of plant protein extract was used for immunoblots. Western analysis was performed with anti-rabbit-RGA antibody (AGRISERA), and immunoblots were incubated with SuperSignal Femto West Substrate (Thermo Fisher Scientific) and detected with a LAS-4000 Mini-image analyzer (Fujifilm).

### Accession numbers

Microarray data have been deposited with the NCBI Gene Expression Omnibus (GEO) under accession number GSE57416. ChIP-seq data have been deposited under accession number GSE54881.

## Additional files

Additional file 1: Table S1. Whole-genome expression profiling experiments comparing the profiles of the genotypes *SVP FLC FRI*, *SVP flc-3 FRI*, *svp-41 FLC FRI* and *svp-41 flc-3 FRI* grown for 2 weeks under short day conditions (SD) or for 2 weeks under SDs and then transferred for 2 days under long days (LD). Additional file 2: Table S2. Whole-genome expression profiling experiments comparing the profiles of the genotypes *SVP FLC FRI*, *svp-41 FLC FRI*, *SVP flc-3 FRI*, and *svp-41 flc-3 FRI* in a transition experiment. Plants were grown for 2 weeks under SD conditions and then transferred for 2 days under LDs. Table describes the effect of treatment for each genotype. Additional file 3: Figure S1. Transcriptome changes in flowering transition from SDs to LDs. **(A)** Comparison of transcriptional profiles in the transition from SDs to LDs in *SVP FLC FRI* wild type, and mutant genotypes *SVP flc-3 FRI*, *svp-41 FLC FRI* and *svp-41 flc-3 FRI.* Genes with fold change >2 and false discovery rate <0.01 were identified as differentially expressed. The heat map represents expression difference values for up-regulated (red) and down-regulated (green) genes in apices (left) and leaves (right). **(B)** Venn diagram for differentially expressed genes during transition from SDs to LDs in the different genotypes. FLC- and SVP-dependent genes are defined as those differentially expressed in the mutants but not in the wild-type genotypes (WT). **(C)** Venn diagram for genes differentially expressed during transition from SDs to LDs in the *FRI FLC SVP* wild-type genotype in both tissues. Additional file 4: Text S1. Microarray data analysis. Rationale of the signalling allocation analysis to identify gene expression clusters. Additional file 5: Figure S2. Different modes of regulation defined by SVP and FLC. **(A)** Flowchart of microarray data analysis for differential gene expression, signalling allocation analysis, and clustering. **(B)** Cartoon describing possible modes of regulations: 1TF dependent; redundant; independent; dependent. The 1TF dependent mode suggests that gene regulation is achieved by binding of only one of the TFs, either SVP or FLC. The redundant mode of regulation suggests that they bind to the same sequence and either of them is functional. The independent mode of regulation suggests that they bind to different DNA regions to additively repress gene expression. The dependent mode suggests that the FLC:SVP protein complex is needed to bind and repress transcription. Cluster names for each mode are also indicated. **(C)** Heat map of coefficients obtained from signalling allocation analysis for genes up-regulated in leaves (199 genes; left) and apices (40 genes; right). Down-regulated genes in mutants were not included in this analysis because most or all of these were assumed to be indirectly regulated. Negative values for the coefficients for FLC and SVP represent positive contributions of each gene to repression of gene expression. For the interaction term, negative values represent that FLC and SVP work cooperatively (dependent); zero, independently and positive, redundant. Letters identify the different clusters found. Additional file 6: Figure S3. Coefficients of the terms FLC, SVP and FLC:SVP in the linear model used for signalling allocation analysis in leaves under SD conditions. **(A)** Heat map of signalling allocation analysis coefficients of all differentially expressed genes in leaves (818 genes). Positive values are represented in red, while negative values are represented in green. Genes having positive coefficients for the single TF (FLC and SVP terms) but a negative coefficient for the complex (term FLC:SVP) are marked with a black line. **(B)** Bar plot of the contribution of each term to gene expression detected in the signalling allocation analysis for genes marked in (A) with the black line (199 genes). **(C)** Average expression of genes located in the group of genes identified in (A). Values are the mean and standard error of these 199 genes. Additional file 7: Figure S4. Transcript levels of genes with expression patterns only affected by either of the two TFs. **(A,B)** Venn diagrams showing genes differentially expressed in *svp-41* (pink), *flc-3* (green) and *svp-41 flc-3* (light-blue) loss of function mutants in leaves (A) and apices (B) as described in Figure 1A. The average fold-change in expression of all genes relative to WT affected by either FLC or SVP and not in double mutant is shown for leaves (A) and apices (B) in the bar plots. Additional file 8: Table S3. Gene expression clusters identified for each tissue. Genes and expression values are described for each cluster. Additional file 9: Figure S5. Gene expression for *GA20OX2*, and signalling allocation analysis for *FT*, *SOC1*, and *GA20OX2*. **(A)** Transcript levels of *GA20OX2* determined by qRT-PCR in apices of *SVP FLC FRI* wild type, and for the mutant genotypes *SVP flc-3 FRI*, *svp-41 FLC FRI* and *svp-41 flc-3 FRI* from 2-week-old seedlings grown under SDs. Data values are represented as log_2_ fold-change relative to wild type. **(B)** Statistical analysis of signalling allocation for individual genes. Data for *FT* were analysed in leaves of plants grown under SDs followed by 2 LDs. *SOC1* and *GA20OX2* were analysed in apices grown under SDs (plain) or SDs followed by 2 LDs (dashed). Asterisks denote *P* <0.01. Additional file 10: Figure S6.
*DIN10* transcription is controlled by FLC and SVP in a complex-dependent manner. **(A,B)** Transcript levels of *DIN10* determined by tilling array and qRT-PCR in leaves of *SVP FLC FRI*, *SVP flc-3 FRI*, *svp-41 FLC FRI* and *svp-41 flc-3 FRI* genotypes for 2-week-old seedlings grown under SDs. Values are represented in log_2_ fold-change relative to wild type (A), or in mean values and stand error of the mean; n = 12 to 14 plants. **(C)** Flowering time of wild-type and *din10* plants under LDs; n = 12 to 14. Values are mean and standard deviation. Asterisks denote statistical significance *P* ≤ 0.05 (*t*-test). **(D)** Phenotypes of wild-type and *din10* mutant plants at flowering time grown under LDs. **(E)** *FT* mRNA levels in 9-, 13- and 15-day-old seedlings of wild type and *din10*. Expression levels relative to PP2A. Additional file 11: Figure S7. Characterization of transgenic plants expressing SVP-GFP translational fusion used for SVP:GFP ChIP-seq. **(A)** Flowering phenotype of plants expressing *pSVP:SVP:GFP* in *svp-41 FLC FRI* and *svp-41 flc-3 FRI* mutant background showing complementation of *svp-41* with the transgene. **(B)** Localization of SVP:GFP under SDs or SDs followed by 5 LDs. The expression pattern in apices was visualized by confocal microscopy demonstrating SVP:GFP responds as SVP wild-type protein. Additional file 12: Text S2. Supplementary methods. Additional file 13: Table S4. High confidence transcription factor binding sites and associated targets of SVP:GFP and FLC in the four genotypes analysed (*SVP:GFP FLC FRI*, *SVP:GFP flc-3 FRI*, *FLC SVP FRI*, *FLC svp-41 FRI*). Additional file 14: Figure S8. Peak annotation in the *Arabidopsis* genome (TAIR10) for ChIP-seq peaks of FLC and SVP proteins in wild-type and mutant genotypes. **(A)** SVP:GFP peak distribution over different genomic features in wild type and *SVP flc-3 FRI* mutant background. **(B)** FLC peak distribution over different genomic features in wild type and *svp-41 FLC FRI* mutant background. Peaks were annotated to chromosomal regions with the Bioconductor Package ChIPpeakAnno. Additional file 15: Table S6. ChIP-seq quantitative comparison for SVP and FLC binding between wild-type (*SVP:GFP FLC FRI*; *FLC SVP FRI*) and mutant (*SVP:GFP flc-3 FRI*; *FLC svp-41 FRI*) genotypes. The table shows the log_2_ of the peak height score change for the list of genomic regions present in both genotypes (ubiquitous (UB)), peaks uniquely identified when both TFs were present (2TF), and peaks uniquely identified in the mutant (1TF). The genomic neighbourhood for any given gene was defined to encompass the 3 kb upstream region and 1 kb downstream region; peak summits overlapping these regions were associated with that gene (Additional file 13). Additional file 16: Table S7. Statistical analysis of differential binding with replicates. The table shows the results of functional PCA on ChIP-seq peak shape (normalized binding) across replicates, followed by a Hotelling's *T* ^*2*^ test over the functional principal component scores on two different treatment conditions (genotypes). The number of PCs to achieve at least 70% of variance, as well as the total variance explained by the chosen components, are indicated. *P*-values were adjusted for multiple comparison using the Benjamini-Hochberg procedure for the control of false discovery rate. Additional file 17: Figure S9. Heat maps and profile plots of the 245 transcription factor binding sites in *SVP flc-3 FRI* in a region ±750 bp around the peak summits in both genotypes. **(A)** SVP binding in the *flc-3* mutant; **(B)** SVP binding in wild type. Summary images above the heat map plot the median profiles. Regions in the heat map in (B) are in the same order as regions in (A). Numbers below the heat map indicate significant differential binding (corrected *P* ≤ 0.05) detected by comparison with the genotype in (A). Additional file 18: Figure S10. Heat maps and profile plots of the 419 transcription factor binding sites in *svp-41 FLC FRI* in a region ±750 bp around the peak summits for both genotypes. **(A)** FLC binding in the *svp-41* mutant genotype; **(B)** FLC binding in wild-type. Summary images above the heat map plot the median profiles. Regions in the heat map in (B) are in the same order as regions in (A). Numbers below the heat map indicate significant differential binding (corrected *P* ≤ 0.05) detected by comparison with the genotype in (A). Additional file 19: Figure S11. Enrichment over background of different CArG-box variants. (CC(A/T)_5_GG, CC(A/T)_6_GG and CC(A/T)_7_GG) in FLC and SVP ChIP-seq regions defined in Figure 3B. The CArG-box with 6-nucleotide long spacer was found significantly enriched in all ChIP regions. Additional file 20: File S1. Position weight matrix for each dataset described in Figure 3B. Additional file 21: Figure S12. Beanplots for the distribution of motif occurrences/instances found in 100 random sets of equal numbers of sequences as in ChIP seq datasets and statistical comparison with ChIP-seq data. **(A,B)** Beanplot distribution of CArG-box, G-box and CE-box motifs identified in FLC ChIP-seq dataset (A) and SVP ChIP-seq dataset (B). Red dots indicate the number of occurrences/instances in ChIP-seq datasets. **(C,D)** Total sequences (under peaks), total occurrences/instances of a motif in those sequences, instances expected by chance (median of distribution of 100 random sets) and *P*-value for observed ocurrences/instances in ChIP-seq datasets for each motif in tabular form. Additional file 22: Figure S13. Binding of FLC and SVP:GFP to flowering- and flower development-related genes *SOC1*, *SEP3* and *AGL16*. **(A)** ChIP-seq enrichment of SVP:GFP and FLC genotypes binding to the *SEP3* and *SOC1* regions. Bar denotes a 5 kb window. **(B)** Local ChIP-seq enrichment of SVP:GFP and FLC genotypes binding to the *AGL16* region in a complex-dependent manner. Bar denotes a 5 kb window. Transcript levels of *AGL16* were determined by qRT-PCR in apices and leaves of *SVP FLC FRI*, *SVP flc-3 FRI*, *svp-41 FLC FRI* and *svp-41 flc-3 FRI* genotypes for 2-week-old seedlings grown under SD conditions. *PP2A* was used as the internal reference. The expression level of each gene in the mutants was normalized to the level in wild type. Error bars represent standard deviation of the mean. Additional file 23: Table S5. List of differentially expressed genes and TF binding in any of the conditions assayed. Additional file 24: Figure S15. GO terms enriched for differentially expressed genes obtained after transcriptome analyses. Only GO terms enriched at a significant level (*P*-value <0.01, X = 2) are represented. Additional file 25: Figure S14. FLC and SVP:GFP binding in a complex-exclusive manner. Local enrichment of SVP:GFP and FLC binding for the 30 peak regions defined to be complex-exclusive after statistical analysis of ChIP-seq data. Peak location and gene annotation are described in the table. Additional file 26: Figure S16. ChIP analysis of FLC (left) and SVP:GFP (right) binding to *DDF1*, *GA2OX8* and *RGL2*. Results are represented as the percentage of input. Additional file 27: Figure S17. Molecular and phenotypic characterization of *GA2OX8* alleles used in this study. **(A)** Model of *GA2OX8* gene structure annotated based on TAIR10. GK-61 F06 and WiscDsLox263B11 insertion sites are marked with a red triangle. Primers used for genotyping each allele are indicated as grey triangles. FLC and SVP common binding site identified by ChIP-seq is depicted with a green bracket. **(B)** Flowering time of GK-61 F06 and WiscDsLox263B11 compared with *fri* Col as control under SDs. Data represent the mean ± standard deviation of 12 to 15 individual plants. Additional file 28: Figure S18. Venn diagrams showing the overlapping set of putative targets for SVP and FLC between this and previous studies. **(A**) Venn diagram for SVP ChIP-seq targets in Gregis *et al*. [4], Tao *et al*. [9] and the results presented in this study. **(B)** Venn diagram for targets of FLC reported in Deng *et al*. [3] and the results presented in this study. **(C)** Comparison between experimental procedures, sequencing technologies, and bioinformatic approaches used for genome-wide identification of SVP and FLC targets in the studies in (A) and (B).

## Abbreviations

AGL: AGAMOUS LIKE
AP: APETALA
bHLH: basic helix-loop-helix
ChIP: chromatin immunoprecipitation
ChIP-on-chip: Chromatin immunoprecipitation combined with DNA microarrays
ChIP-seq: Chromatin immunoprecipitation followed by high-throughput sequencing
COL: CONSTANS-LIKE
DDF1: DWARF AND DELAYED FLOWERING 1
DEG: differentially expressed genes
DIN10: DARK INDUCIBLE 10
FLC: FLOWERING LOCUS
FRI: FRIGIDA
FT: FLOWERING LOCUS T
GA: gibberellin
GA20OX2: GIBBERELLIN 20 OXIDASE 2
GA2OX7: GIBBERELLIN 2-OXIDASE
GFP: green fluorescent protein
GO: Gene Ontology
IDR: irreproducible discovery rate
JAZ: JASMONATE ZIM-domain
LD: long day
MAF3: MADS AFFECTING FLOWERING 3
PCA: principal component analysis
qRT-PCR: real-time quantitative PCR
SD: short day
SEP3: SEPALLATA3
SHP2/AGL5: SHATTERPROOF 2
SOC1: SUPPRESSOR OF OVEREXPRESSION OF CONSTANS1
SVP: SHORT VEGETATIVE PHASE
TEM: TEMPRANILLO
TF: transcription factor
UB: ubiquitous
UTR: untranslated region
WT: wild-type
ZT: Zeitgeber
