# Supplementary figures and images for: Combinatorial activities of SHORT VEGETATIVE PHASE and FLOWERING LOCUS C define distinct modes of flowering regulation in Arabidopsis
Source: Genome Biol. 2015 Feb 11;16(1):31. doi: 10.1186/s13059-015-0597-1 (PMC4378019; doi:10.1186/s13059-015-0597-1)

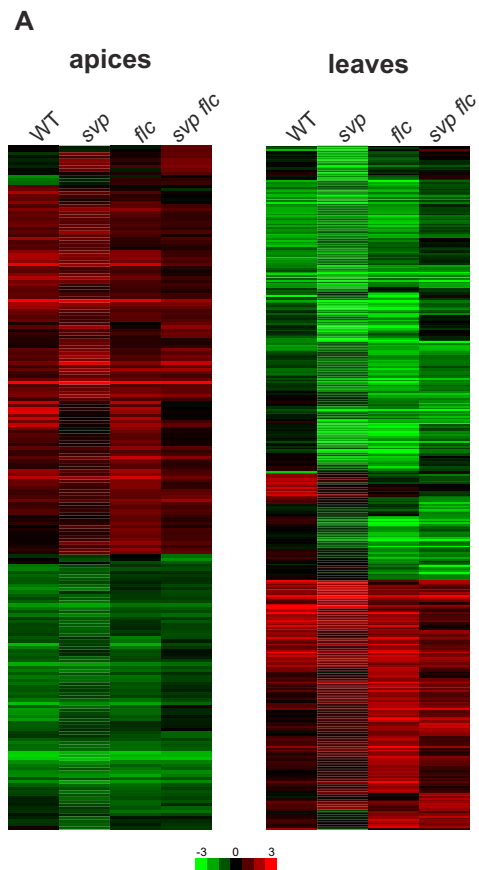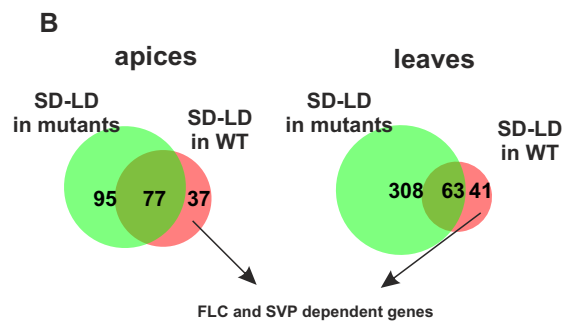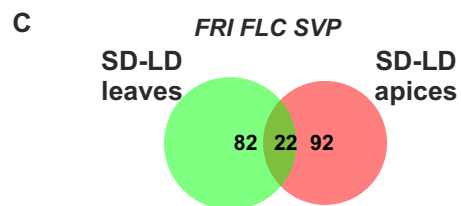

Supplement: Additional file 3: Figure S1. — Transcriptome changes in flowering transition from SDs to LDs. (A) Comparison of transcriptional profiles in the transition from SDs to LDs in SVP FLC FRI wild type, and mutant genotypes SVP flc-3 FRI, svp-41 FLC FRI and svp-41 flc-3 FRI. Genes with fold change >2 and false discovery rate <0.01 were identified as differentially expressed. The heat map represents expression difference values for up-regulated (red) and down-regulated (green) genes in apices (left) and leaves (right). (B) Venn diagram for differentially expressed genes during transition from SDs to LDs in the different genotypes. FLC- and SVP-dependent genes are defined as those differentially expressed in the mutants but not in the wild-type genotypes (WT). (C) Venn diagram for genes differentially expressed during transition from SDs to LDs in the FRI FLC SVP wild-type genotype in both tissues. [file 13059_2015_597_MOESM3_ESM.pdf]

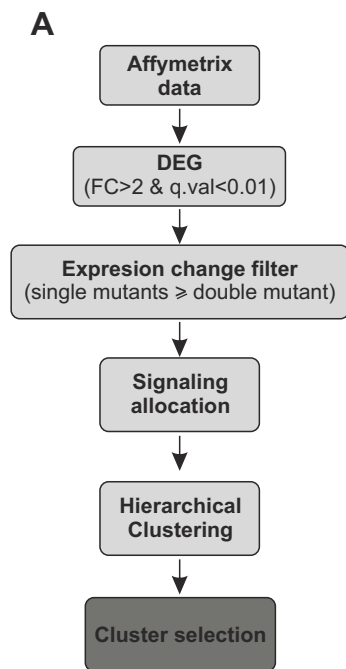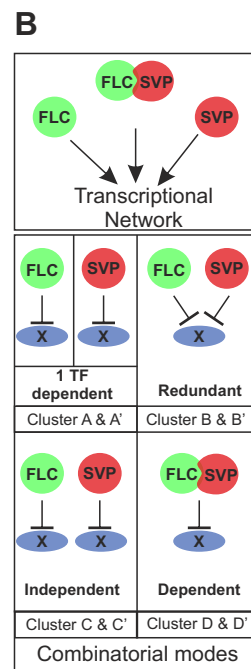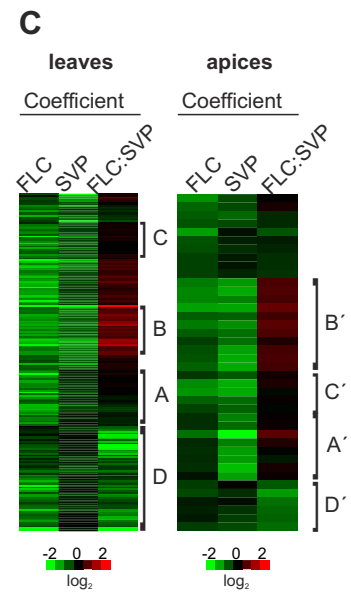

Supplement: Additional file 5: Figure S2. — Different modes of regulation defined by SVP and FLC. (A) Flowchart of microarray data analysis for differential gene expression, signalling allocation analysis, and clustering. (B) Cartoon describing possible modes of regulations: 1TF dependent; redundant; independent; dependent. The 1TF dependent mode suggests that gene regulation is achieved by binding of only one of the TFs, either SVP or FLC. The redundant mode of regulation suggests that they bind to the same sequence and either of them is functional. The independent mode of regulation suggests that they bind to different DNA regions to additively repress gene expression. The dependent mode suggests that the FLC:SVP protein complex is needed to bind and repress transcription. Cluster names for each mode are also indicated. (C) Heat map of coefficients obtained from signalling allocation analysis for genes up-regulated in leaves (199 genes; left) and apices (40 genes; right). Down-regulated genes in mutants were not included in this analysis because most or all of these were assumed to be indirectly regulated. Negative values for the coefficients for FLC and SVP represent positive contributions of each gene to repression of gene expression. For the interaction term, negative values represent that FLC and SVP work cooperatively (dependent); zero, independently and positive, redundant. Letters identify the different clusters found. [file 13059_2015_597_MOESM5_ESM.pdf]

# A

## leaves

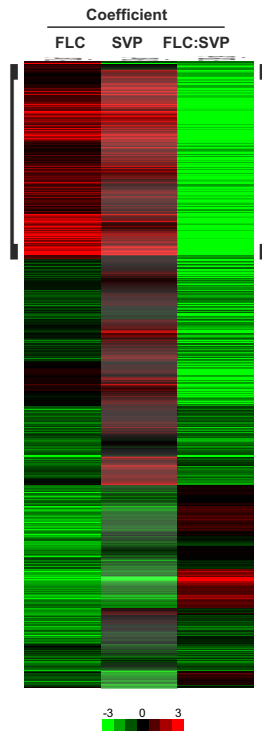

Supplement: Additional file 6: Figure S3. — Coefficients of the terms FLC, SVP and FLC:SVP in the linear model used for signalling allocation analysis in leaves under SD conditions. (A) Heat map of signalling allocation analysis coefficients of all differentially expressed genes in leaves (818 genes). Positive values are represented in red, while negative values are represented in green. Genes having positive coefficients for the single TF (FLC and SVP terms) but a negative coefficient for the complex (term FLC:SVP) are marked with a black line. (B) Bar plot of the contribution of each term to gene expression detected in the signalling allocation analysis for genes marked in (A) with the black line (199 genes). (C) Average expression of genes located in the group of genes identified in (A). Values are the mean and standard error of these 199 genes. [file 13059_2015_597_MOESM6_ESM.pdf]

**A**

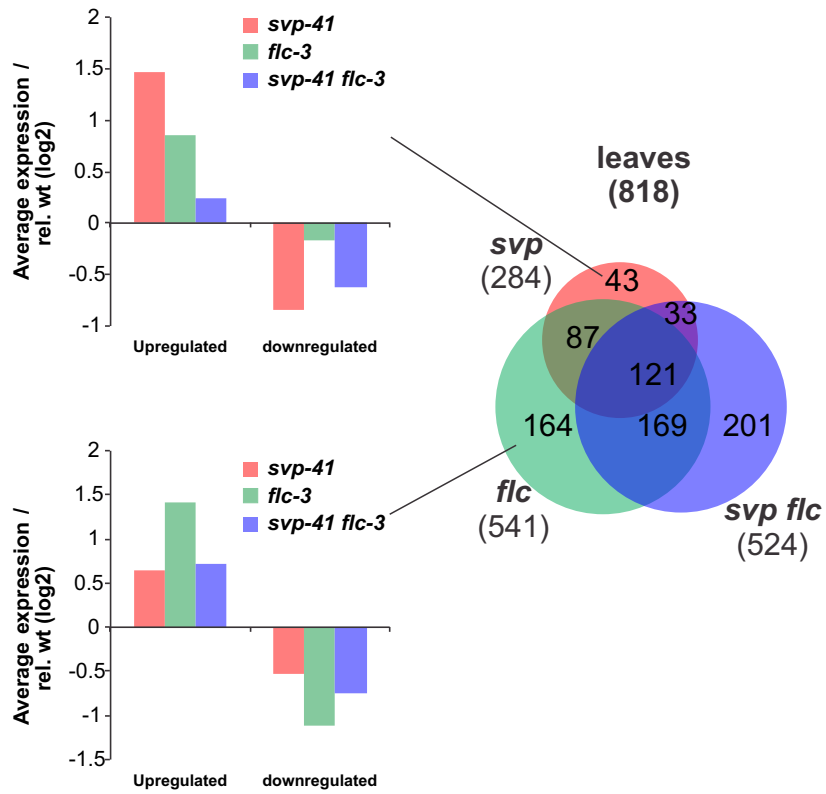

## B

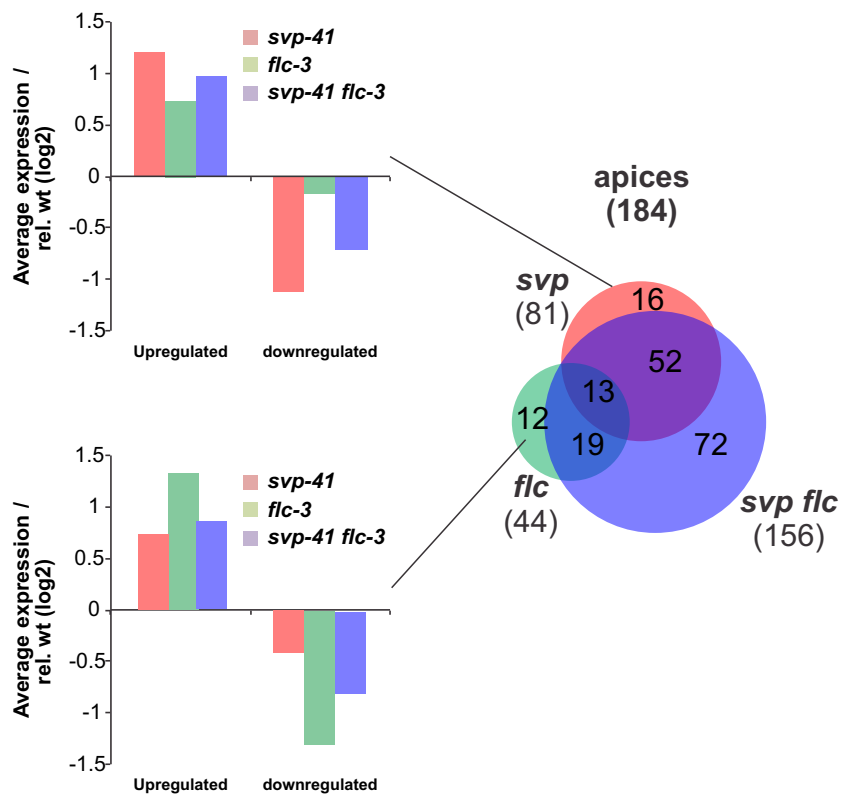

Supplement: Additional file 7: Figure S4. — Transcript levels of genes with expression patterns only affected by either of the two TFs. (A,B) Venn diagrams showing genes differentially expressed in svp-41 (pink), flc-3 (green) and svp-41 flc-3 (light-blue) loss of function mutants in leaves (A) and apices (B) as described in Figure 1A. The average fold-change in expression of all genes relative to WT affected by either FLC or SVP and not in double mutant is shown for leaves (A) and apices (B) in the bar plots. [file 13059_2015_597_MOESM7_ESM.pdf]

**A**

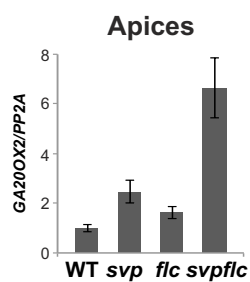

**B**

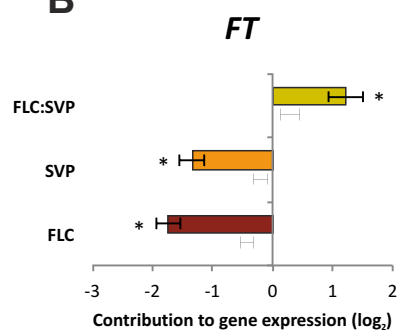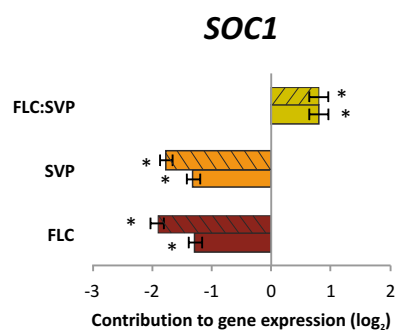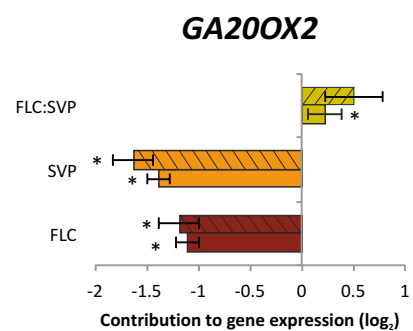

Supplement: Additional file 9: Figure S5. — Gene expression for GA20OX2, and signalling allocation analysis for FT, SOC1, and GA20OX2. (A) Transcript levels of GA20OX2 determined by qRT-PCR in apices of SVP FLC FRI wild type, and for the mutant genotypes SVP flc-3 FRI, svp-41 FLC FRI and svp-41 flc-3 FRI from 2-week-old seedlings grown under SDs. Data values are represented as log2 fold-change relative to wild type. (B) Statistical analysis of signalling allocation for individual genes. Data for FT were analysed in leaves of plants grown under SDs followed by 2 LDs. SOC1 and GA20OX2 were analysed in apices grown under SDs (plain) or SDs followed by 2 LDs (dashed). Asterisks denote P <0.01. [file 13059_2015_597_MOESM9_ESM.pdf]

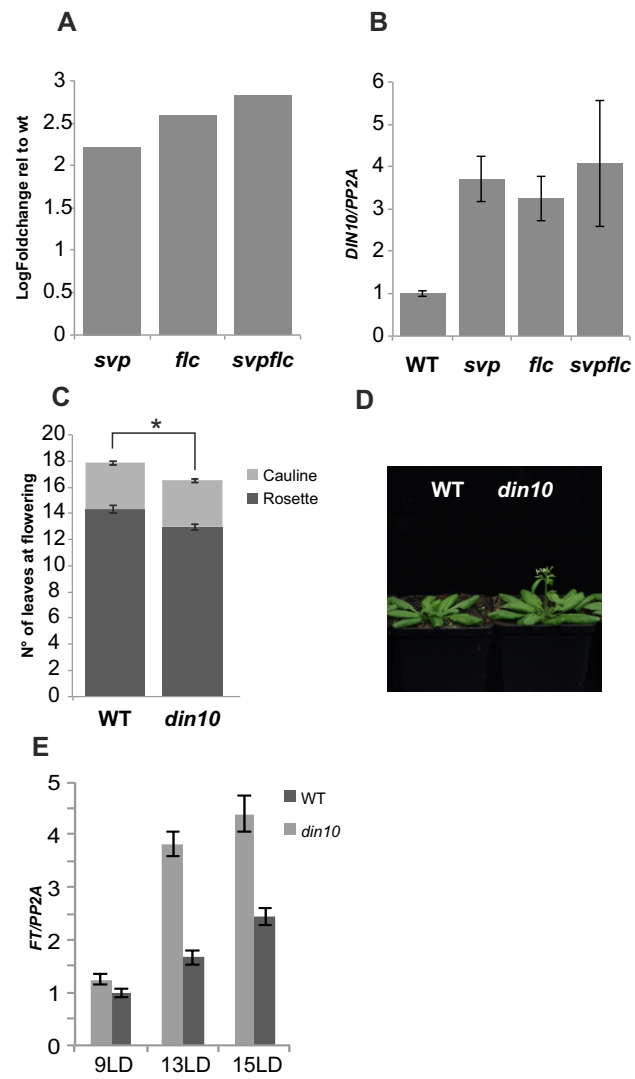

Supplement: Additional file 10: Figure S6. — DIN10 transcription is controlled by FLC and SVP in a complex-dependent manner. (A,B) Transcript levels of DIN10 determined by tilling array and qRT-PCR in leaves of SVP FLC FRI, SVP flc-3 FRI, svp-41 FLC FRI and svp-41 flc-3 FRI genotypes for 2-week-old seedlings grown under SDs. Values are represented in log2 fold-change relative to wild type (A), or in mean values and stand error of the mean; n = 12 to 14 plants. (C) Flowering time of wild-type and din10 plants under LDs; n = 12 to 14. Values are mean and standard deviation. Asterisks denote statistical significance P ≤ 0.05 (t-test). (D) Phenotypes of wild-type and din10 mutant plants at flowering time grown under LDs. (E) FT mRNA levels in 9-, 13- and 15-day-old seedlings of wild type and din10. Expression levels relative to PP2A. [file 13059_2015_597_MOESM10_ESM.pdf]

**A**

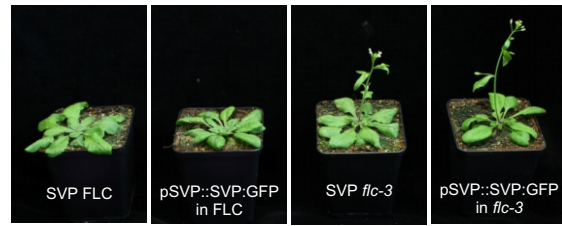

**B**

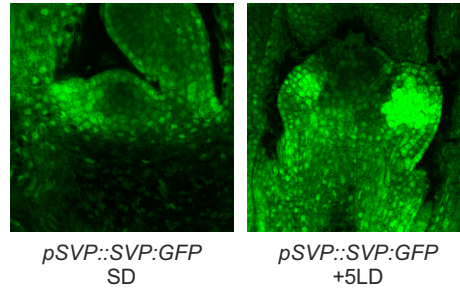

Supplement: Additional file 11: Figure S7. — Characterization of transgenic plants expressing SVP-GFP translational fusion used for SVP:GFP ChIP-seq. (A) Flowering phenotype of plants expressing pSVP:SVP:GFP in svp-41 FLC FRI and svp-41 flc-3 FRI mutant background showing complementation of svp-41 with the transgene. (B) Localization of SVP:GFP under SDs or SDs followed by 5 LDs. The expression pattern in apices was visualized by confocal microscopy demonstrating SVP:GFP responds as SVP wild-type protein. [file 13059_2015_597_MOESM11_ESM.pdf]

**A**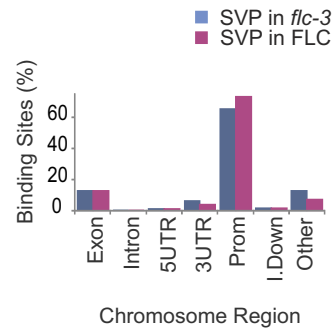**B**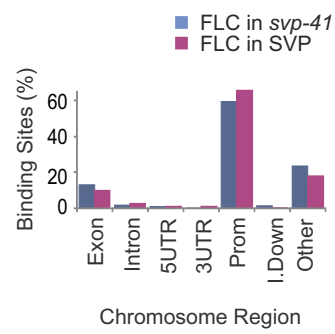

Supplement: Additional file 14: Figure S8. — Peak annotation in the Arabidopsis genome (TAIR10) for ChIP-seq peaks of FLC and SVP proteins in wild-type and mutant genotypes. (A) SVP:GFP peak distribution over different genomic features in wild type and SVP flc-3 FRI mutant background. (B) FLC peak distribution over different genomic features in wild type and svp-41 FLC FRI mutant background. Peaks were annotated to chromosomal regions with the Bioconductor Package ChIPpeakAnno. [file 13059_2015_597_MOESM14_ESM.pdf]

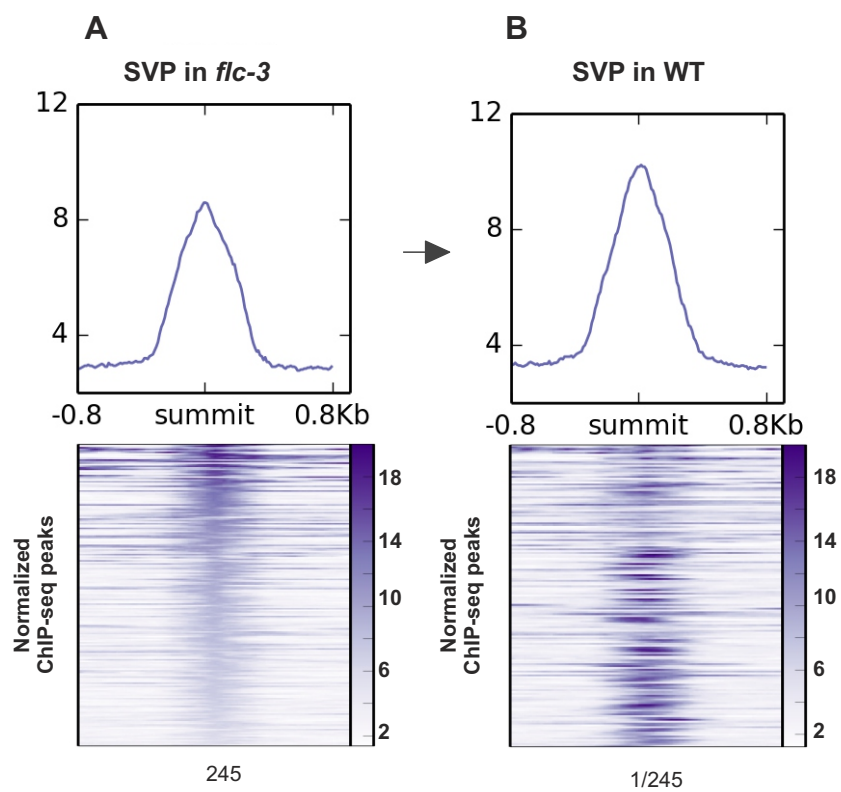

Supplement: Additional file 17: Figure S9. — Heat maps and profile plots of the 245 transcription factor binding sites in SVP flc-3 FRI in a region ±750 bp around the peak summits in both genotypes. (A) SVP binding in the flc-3 mutant; (B) SVP binding in wild type. Summary images above the heat map plot the median profiles. Regions in the heat map in (B) are in the same order as regions in (A). Numbers below the heat map indicate significant differential binding (corrected P ≤ 0.05) detected by comparison with the genotype in (A). [file 13059_2015_597_MOESM17_ESM.pdf]

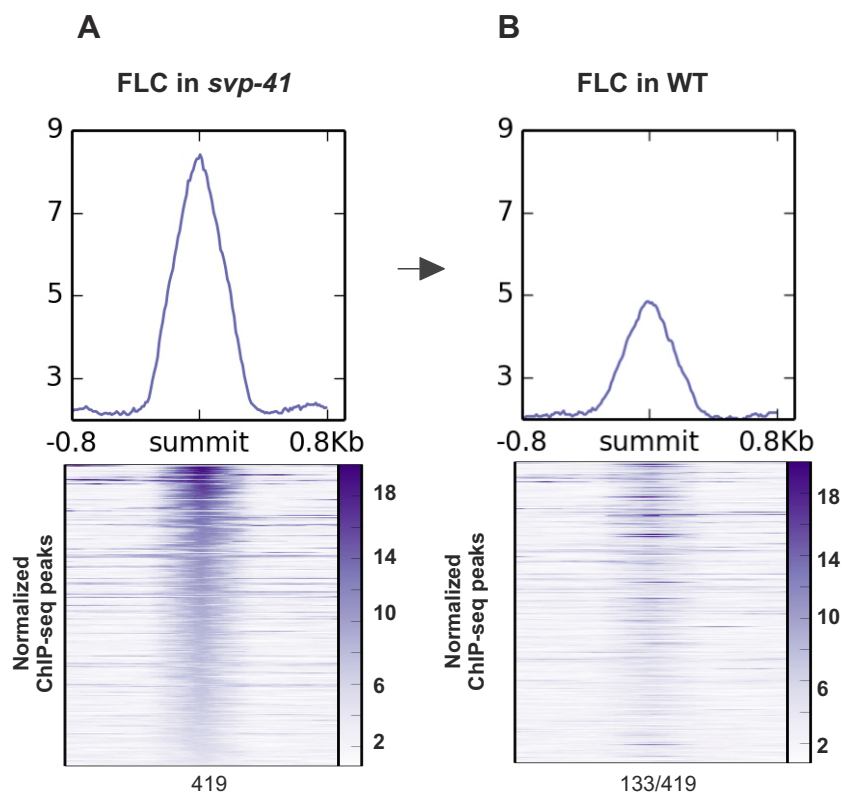

Supplement: Additional file 18: Figure S10. — Heat maps and profile plots of the 419 transcription factor binding sites in svp-41 FLC FRI in a region ±750 bp around the peak summits for both genotypes. (A) FLC binding in the svp-41 mutant genotype; (B) FLC binding in wild-type. Summary images above the heat map plot the median profiles. Regions in the heat map in (B) are in the same order as regions in (A). Numbers below the heat map indicate significant differential binding (corrected P ≤ 0.05) detected by comparison with the genotype in (A). [file 13059_2015_597_MOESM18_ESM.pdf]

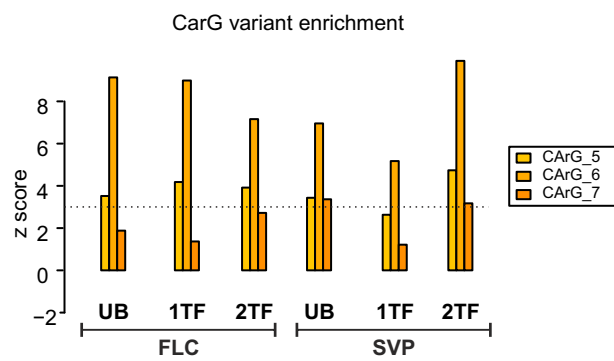

Supplement: Additional file 19: Figure S11. — Enrichment over background of different CArG-box variants. (CC(A/T)5GG, CC(A/T)6GG and CC(A/T)7GG) in FLC and SVP ChIP-seq regions defined in Figure 3B. The CArG-box with 6-nucleotide long spacer was found significantly enriched in all ChIP regions. [file 13059_2015_597_MOESM19_ESM.pdf]

**A**

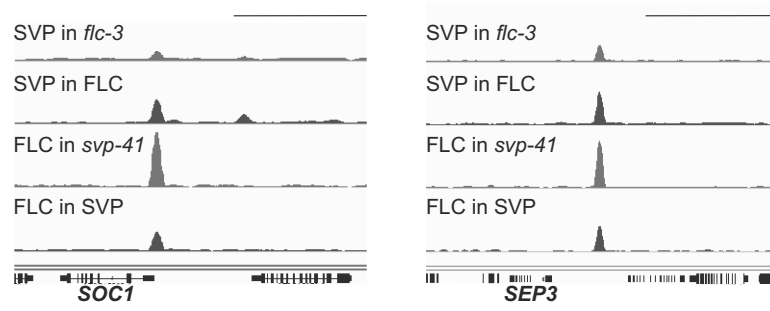

**B**

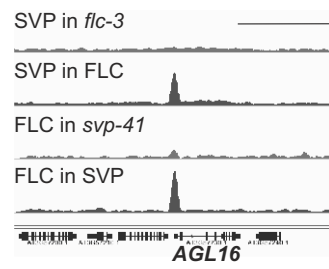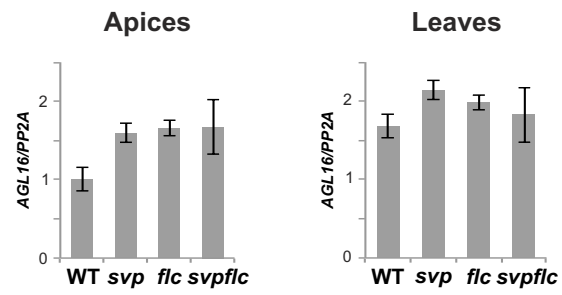

Supplement: Additional file 22: Figure S13. — Binding of FLC and SVP:GFP to flowering- and flower development-related genes SOC1, SEP3 and AGL16. (A) ChIP-seq enrichment of SVP:GFP and FLC genotypes binding to the SEP3 and SOC1 regions. Bar denotes a 5 kb window. (B) Local ChIP-seq enrichment of SVP:GFP and FLC genotypes binding to the AGL16 region in a complex-dependent manner. Bar denotes a 5 kb window. Transcript levels of AGL16 were determined by qRT-PCR in apices and leaves of SVP FLC FRI, SVP flc-3 FRI, svp-41 FLC FRI and svp-41 flc-3 FRI genotypes for 2-week-old seedlings grown under SD conditions. PP2A was used as the internal reference. The expression level of each gene in the mutants was normalized to the level in wild type. Error bars represent standard deviation of the mean. [file 13059_2015_597_MOESM22_ESM.pdf]

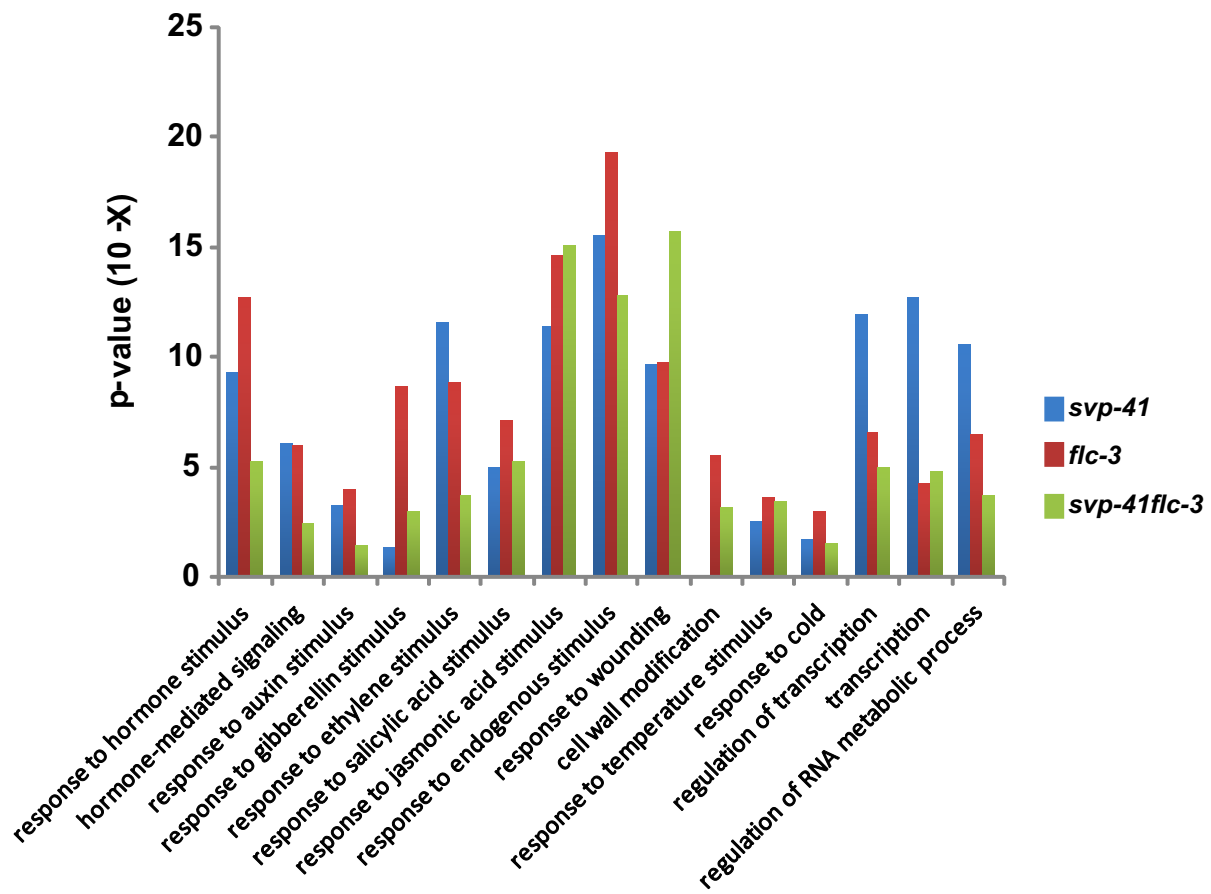

Supplement: Additional file 24: Figure S15. — GO terms enriched for differentially expressed genes obtained after transcriptome analyses. Only GO terms enriched at a significant level (P-value <0.01, X = 2) are represented. [file 13059_2015_597_MOESM24_ESM.pdf]

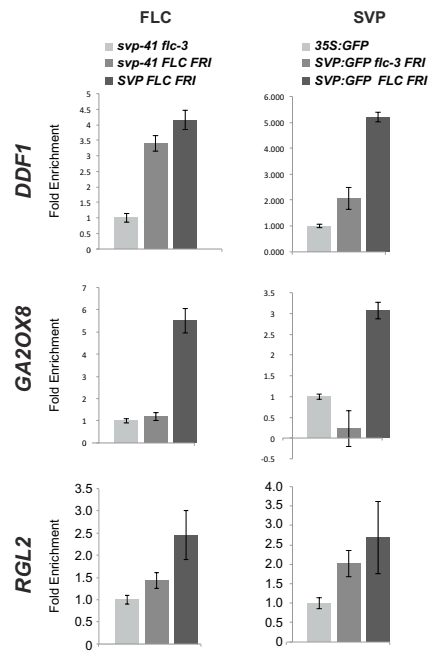

Supplement: Additional file 26: Figure S16. — ChIP analysis of FLC (left) and SVP:GFP (right) binding to DDF1, GA2OX8 and RGL2. Results are represented as the percentage of input. [file 13059_2015_597_MOESM26_ESM.pdf]

**A**

***AT4G21200.1***

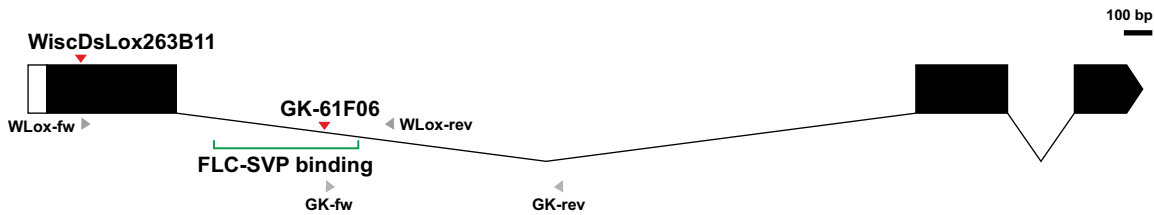

**B**

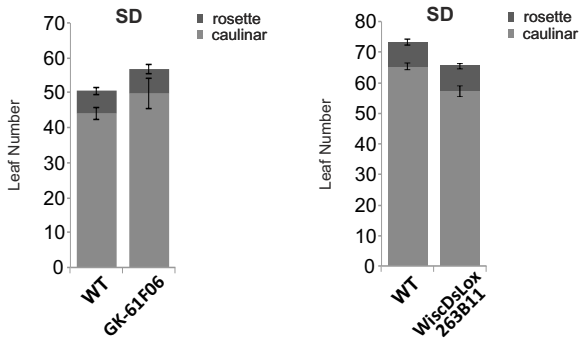

Supplement: Additional file 27: Figure S17. — Molecular and phenotypic characterization of GA2OX8 alleles used in this study. (A) Model of GA2OX8 gene structure annotated based on TAIR10. GK-61 F06 and WiscDsLox263B11 insertion sites are marked with a red triangle. Primers used for genotyping each allele are indicated as grey triangles. FLC and SVP common binding site identified by ChIP-seq is depicted with a green bracket. (B) Flowering time of GK-61 F06 and WiscDsLox263B11 compared with fri Col as control under SDs. Data represent the mean ± standard deviation of 12 to 15 individual plants. [file 13059_2015_597_MOESM27_ESM.pdf]
