# Supplementary material for: Combinatorial activities of SHORT VEGETATIVE PHASE and FLOWERING LOCUS C define distinct modes of flowering regulation in Arabidopsis
Source: Genome Biol. 2015 Feb 11;16(1):31. doi: 10.1186/s13059-015-0597-1 (PMC4378019; doi:10.1186/s13059-015-0597-1)
Supplement: Additional file 4: Text S1. — Microarray data analysis. Rationale of the signalling allocation analysis to identify gene expression clusters. [file 13059_2015_597_MOESM4_ESM.docx]

**Microarray data analysis**

**Rationale of the signaling allocation analysis to identify gene expression clusters**

FLC and SVP control transcription of many genes. FLC and SVP physically interact to form a heterodimer and bind to common sites, therefore the output of the transcriptional network governed by both proteins is determined by the individual action of each TF and by their interaction. In this context interaction includes the physical interaction between them (complex formation) and genetic interactions due to shared DNA binding activity (redundancy or competition). Mutations impairing each TF affect both the individual activity and the interaction. For example, mutations in *SVP* not only disrupt SVP function as a single TF but also that of the interaction FLC:SVP. Therefore, the output of the network is determined by the combined effects of FLC and SVP, and their interaction FLC:SVP. In this way, if the interaction FLC:SVP is significant in controlling gene expression (which is not known so far), the effect of SVP or FLC cannot be estimated by studying *svp-41* or *flc-3* single mutants respectively, but comparisons should include the double mutant *flc-3 svp-41*.

To determine the role of the wild-type TFs and the relationship between them in controlling gene expression in leaf and apex, we quantified genome-wide expression patterns in single and double mutants and fitted these to a linear model (see Methods and [[1](#_ENREF_1)]). We obtained the coefficient for each term of the model, FLC, SVP and FLC:SVP. Interaction terms (or interaction coefficient) could be of three classes: negative, positive or zero. Negative values represent a positive contribution of the complex to gene repression. A negative interaction coefficient applies to cases in which both TFs need to be present to repress expression of genes, for example acting together as a heterodimer. If the coefficient for the FLC:SVP interaction is positive, FLC and SVP function redundantly. The third case is when interaction is zero, indicative of the TFs acting independently. A graphical description is shown in Additional File 5: Figure S2. Then, performing hierarchal clustering on the coefficients calculated for each term, we could identify groups of genes that could be associated with the different cases described in Additional File 5: Figure S2. These different sets of genes defined the clusters used in this study. By looking at the coefficients of the terms in the model for each cluster of genes, we could define the wild-type effect of FLC, SVP and the complex in mastering the transcriptome in different tissues.

REFERENCES

1. Tsuda K, Sato M, Stoddard T, Glazebrook J, Katagiri F: **Network properties of robust immunity in plants.** *PLoS Genet* 2009, **5:**e1000772.
