# Supplementary material for: Combinatorial activities of SHORT VEGETATIVE PHASE and FLOWERING LOCUS C define distinct modes of flowering regulation in Arabidopsis
Source: Genome Biol. 2015 Feb 11;16(1):31. doi: 10.1186/s13059-015-0597-1 (PMC4378019; doi:10.1186/s13059-015-0597-1)
Supplement: Additional file 12: Text S2. — Supplementary methods. [file 13059_2015_597_MOESM12_ESM.docx]

**Combinatorial activities of SHORT VEGETATIVE PHASE and FLOWERING LOCUS C define distinct modes of flowering regulation in Arabidopsis**

Julieta L. Mateos, Pedro Madrigal, Kenichi Tsuda, Vimal Rawat, René Richter,Maida Romera-Branchat, Fabio Fornara, Paweł Krajewski, George Coupland

**Supplementary Methods**

**ChIP-seq data analysis**

We followed recommended guidelines in the analysis of ChIP-seq data for quality control, read mapping, normalization, peak-calling, assessment of reproducibility among biological replicates, post-processing of peaks, and comparison between different treatment conditions [[1](#_ENREF_1)].

***Quality check and read mapping***

Low-quality and duplicated reads in the FASTQ files were filtered out using Parallel-QC 1.0 [[2](#_ENREF_2)]. Low-quality reads were considered as those not having Phred quality scores ≥ 13 (probability that the base called is incorrect ≤ 0.05) in all the bases called. Duplicated reads were also removed to achieve a better specificity (fewer false positive peaks) during the peak calling step of each treatment sample in presence of a control sample [[1](#_ENREF_1), [3](#_ENREF_3)]. Reads kept were then mapped to the *Arabidopsis thaliana* genome (TAIR10) using Bowtie [[4](#_ENREF_4)] version 2.0.2 under default parameters (i.e., reporting the 'best' alignment if multiple mapping locations were found for a read). Then, alignments reported in SAM format were sorted by chromosomal location and converted to indexed binary format (BAM) using SAMtools [[5](#_ENREF_5)].

***Assessment of global reproducibility***

The reproducibility between replicates in ChIP-seq can be measured in two different ways [[1](#_ENREF_1)]: reproducibility of reads, and reproducibility of identified peaks (see next section). Reproducibility between replicates was first assessed using the Pearson Correlation Coefficient (PCC) for each possible pair of replicates both biological and technical, using the genome-wide normalized read (extended to 300 bp) count distribution on a single nucleotide resolution. For this, we used the script ‘correlation.awk’ provided in [[6](#_ENREF_6)]. PCC values were at least 0.96 for all pair-wise comparisons between biological samples, which indicates high similarity between replicates’ binding landscapes [[6](#_ENREF_6)]. Technical replicates showed very high similarity and were merged.

Table 1. Pearson Correlation Coefficient (PCC) ranges for technical and biological replicates.

| **Experimental condition** | **Pearson Corr. Coefficient** |
| --- | --- |
| *SVP::SVP:GFP* *SVP FLC* *FRI* * | 0.960 ≤ PCC ≤ 0.987 |
| *SVP::SVP:GFP* *svp-41 flc-3 FRI* * | 0.970 ≤ PCC ≤ 0.993 |
| *FLC* *FRI SVP* ** | 0.991 ≤ PCC ≤ 0.995 |
| *FLC* *FRI svp-41*** | 0.991 ≤ PCC ≤ 0.992 |

*****= SVP:GFP ChIP-seq using GFP antibody

******= FLC ChIP-seq using FLC anti-serum

***Peak calling***

Peak-calling was performed using MACS [[7](#_ENREF_7), [8](#_ENREF_8)] version 2.0.10, allowing a relaxed *p*-value cut-off (*p*-value≤1e-3, *mfold* range to build the model 2-20, --to-large = TRUE, keep-dup = 1, rest parameters default). Relaxed thresholds are suggested in order to enable the correct computation of IDR values [[9](#_ENREF_9)]. Following the recommendations for the analysis of self-consistency and reproducibility between replicates the four negative control samples were combined into one single control (code for IDR analysis was downloaded from <https://sites.google.com/site/anshulkundaje/projects/idr>, [[10](#_ENREF_10)]). This is also beneficial as control samples with substantially higher number of reads are recommended for peak calling [[1](#_ENREF_1)]. No substantial differences were found when repeating the analysis without pooling the control samples.

Table 2. Peak calling results in MACS

| **Experimental condition** | **#Replicate** | **#Peaks (MACS, *p*-value ≤ 1e-3)** |
| --- | --- | --- |
| *SVP::SVP:GFP* *SVP FLC* FRI * | 1 | 16,973 peaks for 458_CF*** vs control |
|  | 2 | 17,821 peaks for 365_F vs control |
|  | 3 | 17,696 peaks for 365_I vs control |
| *SVP::SVP:GFP* *svp-41 flc-3 FRI* * | 1 | 9,908 peaks for 458_BE*** vs control |
|  | 2 | 36,304 peaks for 365_E vs control |
|  | 3 | 18,347 peaks for 365_H vs control |
| *FLC* *FRI SVP* ** | 1 | 11,352 peaks for 611_A vs control |
|  | 2 | 3,931 peaks for 611_D vs control |
|  | 3 | 2,210 peaks for 611_G vs control |
| *FLC* *FRI svp-41*** | 1 | 6,714 peaks for 611_B vs control |
|  | 2 | 4,890 peaks for 611_E vs control |
|  | 3 | 7,517 peaks for 611_H vs control |

*****= SVP:GFP ChIP-seq using GFP antibody

******= FLC ChIP-seq using FLC anti-serum

*******= Technical replicates C and F, and B and E, respectively, were combined.

***Conservative estimate of reproducible peaks***

To estimate the Irreproducible Discovery Rate (IDR) between replicates (3 pair-wise estimations for each treatment condition) top 16,000 peaks for *SVP::SVP:GFP* *svp-41 FLC* FRI, top 9,900 peaks for *SVP::SVP:GFP* *svp-41 flc-3* FRI, top 2,210 peaks for *41 FLC* *FRI SVP*, and top 4,890 peaks for *FLC* *FRI* *svp-41* (*p*-value ranked) at each biological replicate were submitted for assessment of reproducibility between peaks [[1](#_ENREF_1), [10](#_ENREF_10)]. Note that the discovery of irreproducible peaks is dominated always by the worst sample. For IDR computation using MACS results, we used *p*-values rather than *q*-values as suggested in <https://sites.google.com/site/anshulkundaje/projects/idr> [[9](#_ENREF_9)]. From the overlaps found (at least 1 bp) for each pair-wise assessment, we recorded the number of peaks found passing a threshold of IDR ≤ 5%. Then, a conservative estimated number of candidate TF binding sites was chosen as the maximum number of reproducible peaks found at any comparison [[9](#_ENREF_9)]. No sample was flagged as invalid as the proportion of reproducible peaks was in the order of ~2 in all cases. We kept the maximum number of peaks shown to be reproducible in any pair-wise comparison.

Table 3. Conservative estimate of reproducible peaks

| **Experimental condition** | **#Replicate pairs** | **#Peak overlaps** | **#Reproducible peaks (IDR ≤ 0.05)** | **Max** |
| --- | --- | --- | --- | --- |
| *SVP::SVP:GFP* *SVP FLC* FRI * | 1 & 2 | 5,018 | 235 | 574 |
|  | 1 & 3 | 3,999 | 408 |  |
|  | 2 & 3 | 4,583 | 574 |  |
| *SVP::SVP:GFP* *SVP flc-3* FRI * | 1 & 2 | 1,418 | 229 | 292 |
|  | 1 & 3 | 2,176 | 202 |  |
|  | 2 & 3 | 2,016 | 292 |  |
| *FLC* *FRI SVP* ** | 1 & 2 | 477 | 253 | 409 |
|  | 1 & 3 | 415 | 199 |  |
|  | 2 & 3 | 522 | 409 |  |
| *FLC* *FRI* s*vp-41*** | 1 & 2 | 920 | 443 | 443 |
|  | 1 & 3 | 842 | 398 |  |
|  | 2 & 3 | 864 | 405 |  |

*****= SVP:GFP ChIP-seq using GFP antibody

******= FLC ChIP-seq using FLC anti-serum

***Post-processing of ChIP-seq peaks***

Combining alternative ChIP-seq peak calling methods [[11](#_ENREF_11)], and post-processing ChIP-seq peaks are common practices able to decrease false positive rates [[1](#_ENREF_1), [11](#_ENREF_11)], and has been done before in the analysis of other plant TF ChIP-seq datasets [[12](#_ENREF_12)]. We used the Bioconductor (http://www.bioconductor.org/, [[13](#_ENREF_13)]) package NarrowPeaks v1.4.0 (score of read-enriched regions ≥ 6.0; allowed gap between region = 100 bp; pmaxscor = 0; rest default) on the read-coverage to analyze peak-shape as a post-processing step after general peak calling using functional principal component analysis [[14](#_ENREF_14)]. Reproducible peaks reported by MACS at the previous step were then retained only if they had any overlap with peaks determined by NarrowPeaks.

The final set of peaks considered in the paper is listed at Additional File12: Table S4.

Table 4. Post-processing of ChIP-seq peaks

| **Experimental condition** | **MACS Reproducible peaks** | **NarrowPeaks** | **Filtered out** | **Final set of peaks** |
| --- | --- | --- | --- | --- |
| *SVP::SVP:GFP* *SVP FLC* *FRI* * | 574 | 1104 | 51 | 523 |
| *SVP::SVP:GFP* *SVP flc-3* *FRI* * | 292 | 619 | 46 | 246 |
| *FLC* *FRI SVP* ** | 409 | 1160 | 94 | 315 |
| *FLC* *FRI svp-41* ** | 443 | 1326 | 24 | 419 |

*****= SVP:GFP ChIP-seq using GFP antibody

******= FLC ChIP-seq using FLC anti-serum

***Peak annotation***

Target genes in Additional File12: Table S4 were obtained using the R/Bioconductor package CSAR [[15](#_ENREF_15)] and the genes annotated in TAIR10 (http://www.arabidopsis.org/) as those having a peak summit (location of max. tag enrichment) in a region spanning from 3Kb upstream (start of the gene) to 1Kb downstream (end of the gene) of the final set of peaks called at IDR ≤ 5% after post-processing.

Analysis of peak distribution over exon, intron, enhancer, proximal promoter, 5´ UTR and 3´ UTR was done in R with the ChIPpeakAnno Package [[16](#_ENREF_16)]. Proximal promoter cutoff was set as 3kb, and immediate promoter cutoff at 1kb. Peaks that reside downstream over immediate downstream cutoff from gene end or upstream over proximal promoter cutoff from gene start were classified as enhancers.

***Overlap between peak regions***

Overlaps between peaks were obtained using BEDtools [[17](#_ENREF_17)]. 144 out of 523 peaks (27.5%) in *SVP::SVP:GFP* *SVP FLC* *FRI* overlap (at least 1 bp) with any of the 246 ChIP-seq peaks in *SVP::SVP:GFP* *SVP flc-3* *FRI*. 148 out of 246 peaks (60.2%) in *SVP::SVP:GFP* *SVP flc-3 FRI* overlap with any of the 523 ChIP-seq peaks in *SVP::SVP:GFP* *SVP FLC* *FRI*.

183 out of 315 peaks (58.1%) in *FLC* *FRI SVP* overlap (at least 1 bp) with any of the 419 ChIP-seq peaks in *FLC* *FRI svp-41*. 175 out of 419 peaks (41.8%) in *FLC* *FRI svp-41* overlap (at least 1 bp) with any of the 315 ChIP-seq peaks in *FLC* *FRI SVP*.

***Quantitative analysis of peak height change***

Once a set of confident peaks at each treatment condition have been determined, it is suggested to consider 'all hits' to adequately capture the protein's affinity (peak height) in a genomic region to better quantify differences in TF binding [[1](#_ENREF_1)]. Therefore, we put duplicated reads back at this step.

First, we divided the consensus set of binding regions for the 2 SVP backgrounds (*SVP::SVP:GFP* *SVP FLC* *FRI* and *SVP::SVP:GFP* *SVP flc-3* *FRI*) into 3 different set of peaks referred as UB, 2TF and 1TF. UB set (ubiquitous) are bound regions encountered in the two backgrounds (spanning the global region: union -not intersection-), 2TF (peaks present in the wild-type background, where 2 TF are active) and 1TF (peaks present in mutant background where 1 transcription factor is active) correspond to the regions described that does not overlap in at least 1bp with any peak called in the other background. We did the same for *FLC* *FRI SVP* and *FLC* *FRI svp-41.*

Secondly, we scored each peak region as described in [[6](#_ENREF_6)] for the quantitative comparison of ChIP-seq samples. Quantile normalization was not used because we did not have similar number of binding sites in the two conditions under comparison, and also because the same antibody (anti-GFP) was used and therefore the signal-to-noise ratio was considered to be comparable across genotypes (for FLC or SVP, respectively). As same control was used in wild-type and mutant conditions, control sample scores were not considered. Figure 3C plot the above mentioned scores for the regions. Additional File 15: Table S6 contains the scores.

***Shape-based differential binding analysis***

To further study the differential binding between SVP and FLC, we performed a dimensionality reduction analysis in the peak shapes for the four experimental conditions using a functional version of PCA, and tested whether significant differences exist between the PCA scores of a binding site, and the corresponding normalized read-enrichment in the other three conditions. The analysis was done using the function ‘narrowpeaksDiff’ in the Bioconductor package NarrowPeaks (<http://www.bioconductor.org/>, [[14](#_ENREF_14)]). We studied the peak shape (normalized coverage) ±750bp around peak summits. We employed one principal component if the proportion of variation explained at a peak region was <70%, and two components otherwise. Peak-shapes where smoothed using a linear combination of 15 B-spline basis functions before PCA analysis. To avoid biases in the analysis we excluded peaks with length longer than 4Kb. Although many global quantitative differences were observed in the quantitative analysis of peak height change in the aggregated normalized read coverage (Figure 3C; Additional File 15: Table S6), differential binding analysis penalized the significance for the differences between the biological replicates at the same condition, thus uncovering robust differential binding.

Values in Additional File 16: Table S7 report the *p*-values (multivariate analysis of variance in the PCA space, Hotelling’s T^2^ test) calculated for an experiment ‘A’ changing with respect to read-enrichment in condition ‘B’. The chi square approximation was used in the Hotelling’s T^2^ test to relax the assumption of data normality (f=”chi” in function Hotelling’s T2, R package ICSNP). To control for multiple testing problem we corrected the *p*-values using Benjamini-Hochberg adjustment [[18](#_ENREF_18)]. Genomic regions were declared as significantly different if Benjamini-Hochberg corrected p≤0.05.

The plots and heatmaps of the transcription factor binding sites in a region +/- 750 bp around the summits in in Figures 2D-E, 3A, Additional File 17: Figure S9, Additional File 18: Figure S10 were generated using deepTools-1.5.3 (<https://github.com/fidelram/deepTools>, [[19](#_ENREF_19)]). Regions were sorted in descendent order by the maximum of the non-overlapping median bin calculated over the regions’ length. Summary images above the heatmaps represent the median profile. In Figure 3A, the heatmaps for the mutant condition (right) plots the binding intensity in the same regions and order than in wild-type (left). Values below the heatmaps indicate the number of significant differentially binding events (Benjamini-Hochberg corrected *p*-value≤0.05) detected in that pair-wise comparison. Missing data was considered as zero. Any region containing an intensity value >20 was not considered.

***Visualization***

IGV genome browser [[20](#_ENREF_20)] captures in Figures 5-6 and Additional File 22: Figure S13, Additional File 23: Figure S14 visualize bigWig format files generated for *FLC* FRI *SVP*, *FLC* FRI *svp-41*, *SVP::SVP:GFP* *SVP FLC* FRI and *SVP::SVP:GFP* *SVP flc-3* FRI and controls. The reads mapped at both DNA strands from 5' to 3' direction were extended to a length of 300 bp, and the read count at each genomic position was normalized to the library size and per million reads.

| *SOC1* | xSOC (+) fwB | ATGGGAGGGAAAAAGATGTGT |
| --- | --- | --- |
|  | xSOC (+) reB | TGGTAATGGTGTTTGTGAAACC |
|  | SOC1--ChIP5-SVP | TGGACGCTTGAAACCTCATCCT |
| *SEP3* | xSEP3 (+) reA | AGATGAGAATCGGACGGCT |
|  | xSEP3 (+) fwA | TCTATTTTGGGTAACGAGGTCC |
|  | xSEP3 (-) reC | CATGTTGATAAATTCAGTTTG |
|  | xSEP3 (-) fwC | CAGAATAAAGCAATTACGTC |
| *RVE2* | xRVE2 (+) _fwA | ACAACTGTCTCAAAATGAAAGAGGA |
|  | xRVE2 (+) _revA | CACTTTTGTGCTGACTAGGCAAT |
| *RGL2* | xRGL2_(+) fwA | AGCATGCACCATGTGACTTC |
|  | xRGL_(+) revA | GTATGGTCCCAATCGCAGCT |
| *AGL19* | xAGL19_(+) fwA | TATGAGCTGGCAACGGACAAG |
|  | xAGL19_r(+) evA | CAGATCCGGTGTCCGTCAAA |
| *JAZ6* | xJAZ6 (+) fwA | AGGACACGTGAAGTATTGTTATGTG |
|  | xJAZ6 (+) reA | GGCCTATGAAGTATGAACGCTATAA |
| *AGL16* | xAGL16 (+) fwA | TGCCATGTGTCAAAACATAACAAGC |
|  | xAGL16 (+) reA | GAGATGTGGTTTTGTTGATCGAAAG |
| *GA2OX8* | xGA2ox8_(+) fwA | TCCCCATATCTCATGCGTTTCT |
|  | xGA2ox8_(+) revA | ACATGCCAACTTGCTATCCCA |
|  | xGA2ox8_(-) fwB | AGACTGACCGGATTGTGGTA |
|  | xGA2ox8_(-) RevB | ATCCGGTTGGATTAGCTCGG |
| *DDF1* | xDDF1 (+) fw | TCCCACGTGTTACTCACAGCT |
|  | xDDF1 (+) rev | TGGGACCAAAATATCCATT |
|  | xDDF1 (-) fw | CAACTGAACGATCAATGTGG |
|  | xDDF1 (-) rev | CTTGCACATAACATGTGGTG |

***List of primers used for ChIP-qPCR***

**List of primers used for qRT-PCR**

| *SVP* | SVP-RT-fw | GAAGGACAGTCGTCGGAGTC |
| --- | --- | --- |
|  | SVP-RT-re | GCCTCTTCCATAGGCAGAAA |
| *JAZ6* | JAZ6 RT-PCR For | AAAATTCGATCTCAAAGGACAAC |
|  | JAZ6 RT-PCR Rev | GCTTTTGTCAAGATTCATGTGACTC |
| *DIN10* | DIN10 RT-PCR For | CTTCTTCGCTTTCTGGCATTG |
|  | DIN10 RT-PCR Rev | CGAACCGCCGGTTTAATCGT |
| *AGL16* | AGL16 RT-PCR For | GATTTCTCCAGCTCCAGCATGA |
|  | AGL16 RT-PCR Rev | GGTGAACGAGATTCCCCTCTC |
| *GA2OX8* | MR94-OX8qPCRfor | CACTTCTATCCGGCAGCTTT |
|  | MR95-OX8qPCrrev | GCAAGAACCTCTGCCAACAT |
| *RGL2* | RGL2_fw | CCAAAACCACTACCAGCTTCTC |
|  | RGL2_rev | CAGCCATCTCAGAAGATCGAAC |
| *AGL19* | *AGL19_fw* | AGCAAGCGAGAGACGAAACA |
|  | *AGL19_rev* | AGCTCCTCGATGGAACATGC |
| *RVE2* | RVE2_Rev | CTGAATCAGCTTGGATCCGGTTAG |
|  | RVE2_Fw | CGAAGCCATGCGCAGAAGTT |
| *DDF1* | DDF1_fw | CGCGATCGTATATGGAAGAC |
|  | DDF1_rev | AACTTCTAACGCCTGGGACA |
| *FT* | FT-qRT-F | CGAGTAACGAACGGTGATGA |
|  | FT-qRT-R | CGCATCACACACTATATAAGTAAAACA |
| *PP2A* | PP2A-fw | CAGCAACGAATTGTGTTTGG |
|  | PP2A-rev | AAATACGCCCAACGAACAAA |
| *UBC21* | UBC21-fw | TCCTCTTAACTGCGACTCAGG |
|  | UBC21-rev | GCGAGGCGTGTATACATTTG |

**List of primers used for genotyping**

| *SVP* | SVP-2F | GATTTTGGATTTTTGACCCACT |
| --- | --- | --- |
|  | SVP-3R | TTAGTCGGTGGCTCTTGTCC |
| *FLC* | AtFLCPro-F | TCATGCGGTACACGTGGCAA |
|  | AtFLCex1-R | TCGCCGGAGGAGAAGCTGTA |
| *DIN10* | DIN10 RT-PCR For | CTTCTTCGCTTTCTGGCATTG |
|  | DIN10RT-PCR Rev | CGAACCGCCGGTTTAATCGT |
| *FRI* | AtFRIex1-F | TTGATAAGGATGAGTGGTTCGA |
|  | AtFRIint1-R | TGTCAACAAAAGGAACCACCTT |
| *GA2OX8* | MR82-617F06ox83 | CTATATGTTTCTAGATCGTACC |
|  | MR83-617F06ox85 | GGCATGTGAAATACAATGAATA |

|  |  |
| --- | --- |

**REFERENCES**

1. Bailey T, Krajewski P, Ladunga I, Lefebvre C, Li Q, Liu T, Madrigal P, Taslim C, Zhang J: **Practical Guidelines for the Comprehensive Analysis of ChIP-seq Data.** *PLoS Comput Biol* 2013, **9:**e1003326.

2. Zhou Q, Su X, Wang A, Xu J, Ning K: **QC-Chain: fast and holistic quality control method for next-generation sequencing data.** *PloS one* 2013, **8:**e60234.

3. Chen Y, Negre N, Li Q, Mieczkowska JO, Slattery M, Liu T, Zhang Y, Kim TK, He HH, Zieba J, et al: **Systematic evaluation of factors influencing ChIP-seq fidelity.** *Nature methods* 2012, **9:**609-614.

4. Langmead B, Trapnell C, Pop M, Salzberg SL: **Ultrafast and memory-efficient alignment of short DNA sequences to the human genome.** *Genome biology* 2009, **10:**R25.

5. Li H, Handsaker B, Wysoker A, Fennell T, Ruan J, Homer N, Marth G, Abecasis G, Durbin R: **The Sequence Alignment/Map format and SAMtools.** *Bioinformatics* 2009, **25:**2078-2079.

6. Bardet AF, He Q, Zeitlinger J, Stark A: **A computational pipeline for comparative ChIP-seq analyses.** *Nat Protoc* 2012, **7:**45-61.

7. Feng J, Liu T, Qin B, Zhang Y, Liu XS: **Identifying ChIP-seq enrichment using MACS.** *Nat Protoc* 2012, **7:**1728-1740.

8. Zhang Y, Liu T, Meyer CA, Eeckhoute J, Johnson DS, Bernstein BE, Nusbaum C, Myers RM, Brown M, Li W, Liu XS: **Model-based analysis of ChIP-Seq (MACS).** *Genome biology* 2008, **9:**R137.

9. Landt SG, Marinov GK, Kundaje A, Kheradpour P, Pauli F, Batzoglou S, Bernstein BE, Bickel P, Brown JB, Cayting P, et al: **ChIP-seq guidelines and practices of the ENCODE and modENCODE consortia.** *Genome Res* 2012, **22:**1813-1831.

10. Li D, Brown J, Huang H, Bickel P: **Measuring reproducibility of high-throughput experiments.** *Annals of Applied Statatistics* 2011, **5:**1752-1779.

11. Schweikert C, Brown S, Tang Z, Smith PR, Hsu DF: **Combining multiple ChIP-seq peak detection systems using combinatorial fusion.** *BMC Genomics* 2012, **13 Suppl 8:**S12.

12. Wuest SE, O'Maoileidigh DS, Rae L, Kwasniewska K, Raganelli A, Hanczaryk K, Lohan AJ, Loftus B, Graciet E, Wellmer F: **Molecular basis for the specification of floral organs by APETALA3 and PISTILLATA.** *Proc Natl Acad Sci U S A* 2012, **109:**13452-13457.

13. Gentleman RC, Carey VJ, Bates DM, Bolstad B, Dettling M, Dudoit S, Ellis B, Gautier L, Ge Y, Gentry J, et al: **Bioconductor: open software development for computational biology and bioinformatics.** *Genome biology* 2004, **5:**R80.

14. Madrigal P, Krajewski P: **NarrowPeaks: Shape-based Analysis of Variation in ChIP-Seq using Functional PCA. R package version 1.9.4.**; 2013. [[http://www.bioconductor.org/](http://www.bioconductor.org/)]

15. Muino JM, Kaufmann K, van Ham RC, Angenent GC, Krajewski P: **ChIP-seq Analysis in R (CSAR): An R package for the statistical detection of protein-bound genomic regions.** *Plant Methods* 2011, **7:**11.

16. Zhu LJ, Gazin C, Lawson ND, Pages H, Lin SM, Lapointe DS, Green MR: **ChIPpeakAnno: a Bioconductor package to annotate ChIP-seq and ChIP-chip data.** *BMC Bioinformatics* 2010, **11:**237.

17. Quinlan AR, Hall IM: **BEDTools: a flexible suite of utilities for comparing genomic features.** *Bioinformatics* 2010, **26:**841-842.

18. Benjamini Y, Hochberg Y: **Controlling the False Discovery Rate: A Practical and Powerful Approach to Multiple Testing.** *Journal of the Royal Statistical Society Series B (Methodological)* 1995, **57:**289–300.

19. Ramirez F, Dundar F, Diehl S, Gruning BA, Manke T: **deepTools: a flexible platform for exploring deep-sequencing data.** *Nucleic Acids Res* 2014, **42:**W187-191.

20. Thorvaldsdottir H, Robinson JT, Mesirov JP: **Integrative Genomics Viewer (IGV): high-performance genomics data visualization and exploration.** *Briefings in bioinformatics* 2013, **14:**178-192.
