# Supplementary material for: Combinatorial activities of SHORT VEGETATIVE PHASE and FLOWERING LOCUS C define distinct modes of flowering regulation in Arabidopsis
Source: Genome Biol. 2015 Feb 11;16(1):31. doi: 10.1186/s13059-015-0597-1 (PMC4378019; doi:10.1186/s13059-015-0597-1)
Supplement: Additional file 21: Figure S12. — Beanplots for the distribution of motif occurrences/instances found in 100 random sets of equal numbers of sequences as in ChIP seq datasets and statistical comparison with ChIP-seq data. (A,B) Beanplot distribution of CArG-box, G-box and CE-box motifs identified in FLC ChIP-seq dataset (A) and SVP ChIP-seq dataset (B). Red dots indicate the number of occurrences/instances in ChIP-seq datasets. (C,D) Total sequences (under peaks), total occurrences/instances of a motif in those sequences, instances expected by chance (median of distribution of 100 random sets) and P-value for observed ocurrences/instances in ChIP-seq datasets for each motif in tabular form. [file 13059_2015_597_MOESM21_ESM.pdf]

A

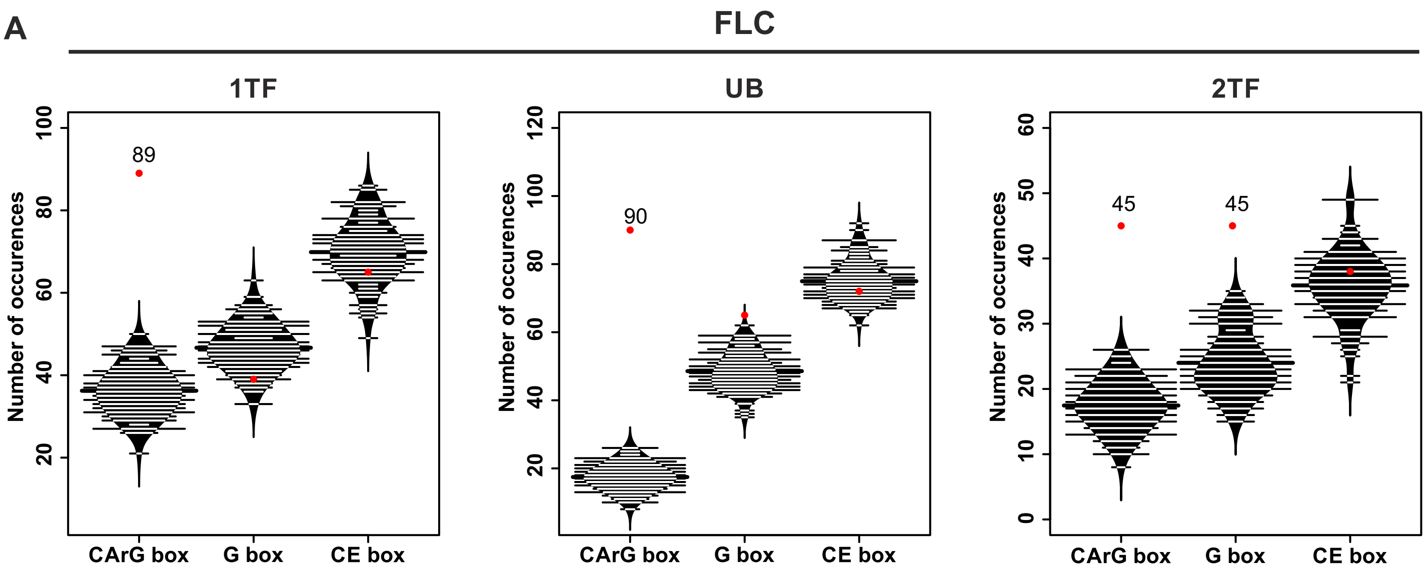

B

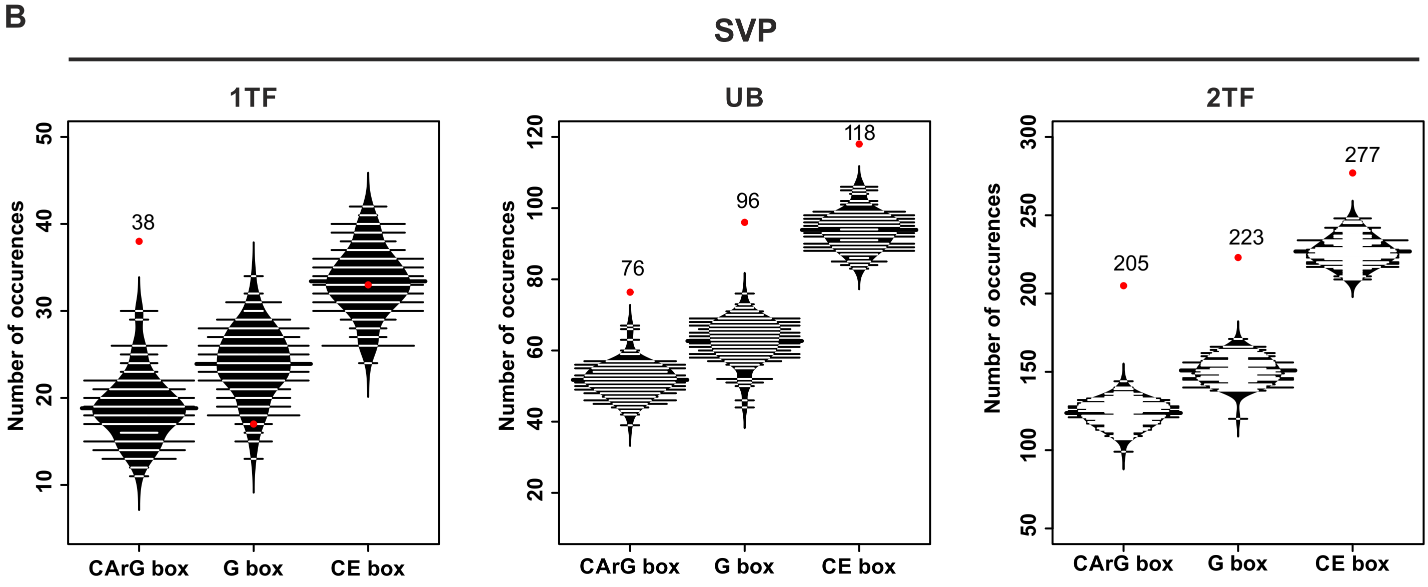

C

FLC

1TF

Cis element	Total Sequences	Occurrences	Occurrences by chance (100 random sets)	P-value
CArG box	244	89	36(median)	2.5e-19
Gbox	244	39	46.5(median)	0.17
CE box	244	65	70(median)	0.5

UB

Cis element	Total Sequences	Occurrences	Occurrences by chance (100 random sets)	P-value
CArG box	175	90	38(median)	6.5e-20
Gbox	175	65	48(median)	0.003
CE box	175	72	75(median)	0.6

2TF

Cis element	Total Sequences	Occurrences	Occurrences by chance (100 random sets)	P-value
CArG box	132	45	17(median)	8.0e-13
Gbox	132	45	23(median)	1.05e-5
CE box	132	38	36median)	0.6

D

SVP

1TF

Cis element	Total Sequences	Occurrences	Occurrences by chance (100 random sets)	P-value
CArG box	98	38	18.5(median)	2.2e-7
Gbox	98	17	25(median)	0.6
CE box	98	33	33(median)	0.9

UB

Cis element	Total Sequences	Occurrences	Occurrences by chance (100 random sets)	P-value
CArG box	144	76	52(median)	1.6e-7
Gbox	144	96	36(median)	8.0e-9
CE box	144	118	94.5(median)	1.5e-6

2TF

Cis element	Total Sequences	Occurrences	Occurrences by chance (100 random sets)	P-value
CArG box	379	205	125(median)	0
Gbox	379	223	151(median)	3.2e-17
CE box	379	273	226.5(median)	3.3e-7
