# Supplementary material for: Combinatorial activities of SHORT VEGETATIVE PHASE and FLOWERING LOCUS C define distinct modes of flowering regulation in Arabidopsis
Source: Genome Biol. 2015 Feb 11;16(1):31. doi: 10.1186/s13059-015-0597-1 (PMC4378019; doi:10.1186/s13059-015-0597-1)
Supplement: Additional file 25: Figure S14. — FLC and SVP:GFP binding in a complex-exclusive manner. Local enrichment of SVP:GFP and FLC binding for the 30 peak regions defined to be complex-exclusive after statistical analysis of ChIP-seq data. Peak location and gene annotation are described in the table. [file 13059_2015_597_MOESM25_ESM.pdf]

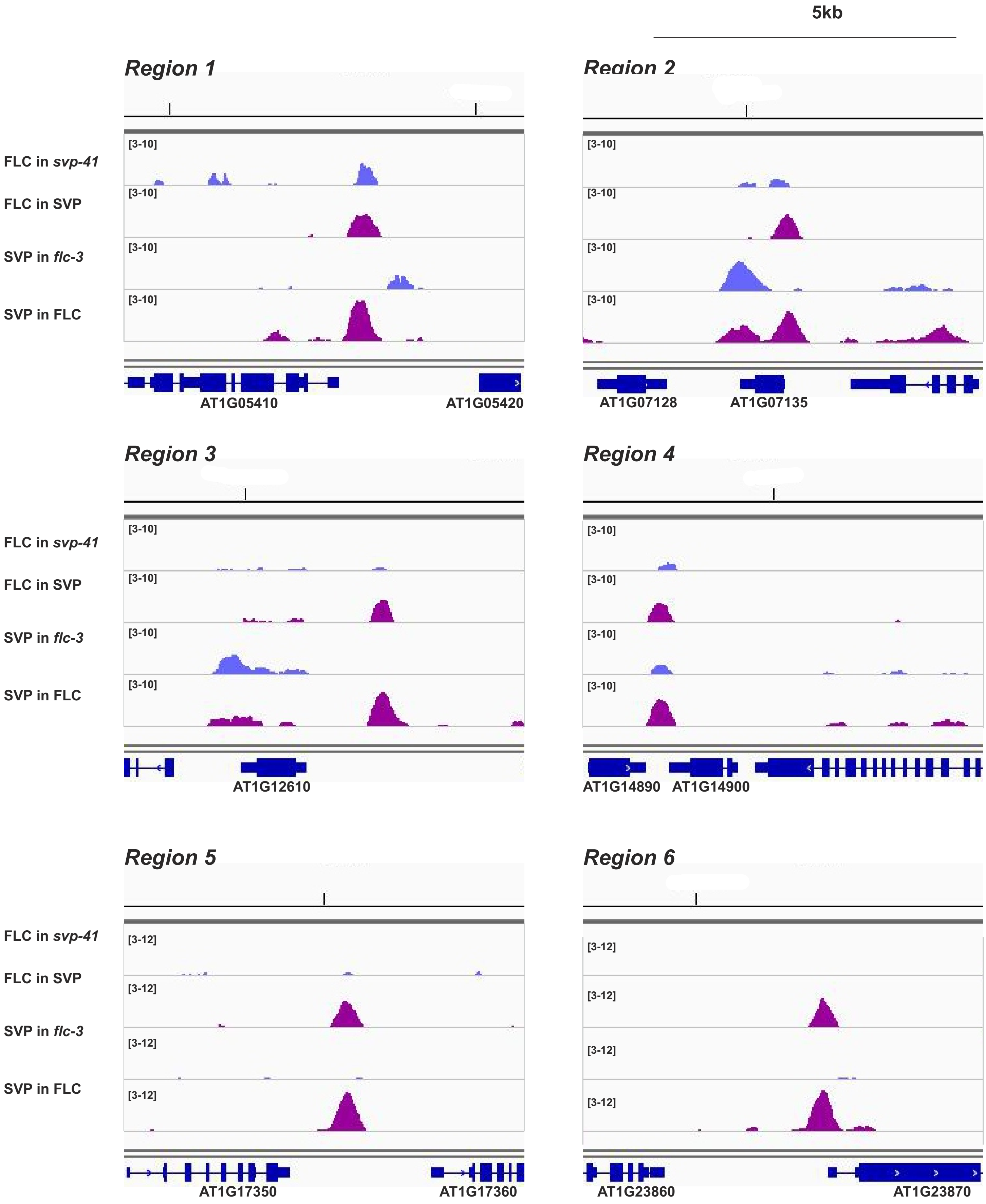

5kb
Region 1
Region 2
Region 3
Region 4
Region 5
Region 6
FLC in svp-41
FLC in SVP
SVP in flc-3
SVP in FLC
[3-10]
[3-12]
AT1G05410
AT1G05420
AT1G07128
AT1G07135
AT1G12610
AT1G14890
AT1G14900
AT1G17350
AT1G17360
AT1G23860
AT1G23870

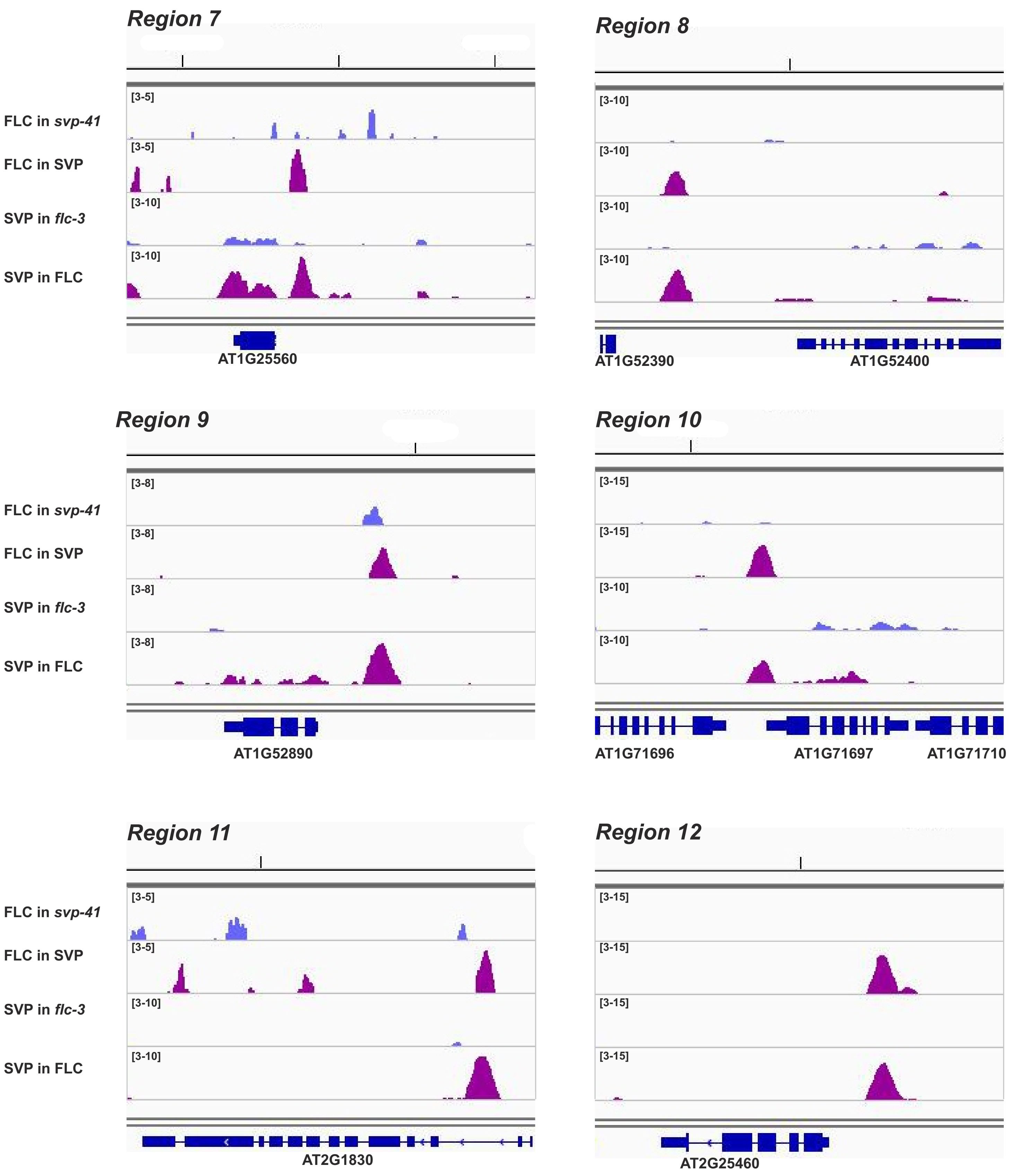

Region 7
FLC in svp-41
FLC in SVP
SVP in flc-3
SVP in FLC
[3-5]
[3-5]
[3-10]
[3-10]
AT1G25560
Region 8
[3-10]
[3-10]
[3-10]
[3-10]
AT1G52390
AT1G52400
Region 9
FLC in svp-41
FLC in SVP
SVP in flc-3
SVP in FLC
[3-8]
[3-8]
[3-8]
[3-8]
AT1G52890
Region 10
[3-15]
[3-15]
[3-10]
[3-10]
AT1G71696
AT1G71697
AT1G71710
Region 11
FLC in svp-41
FLC in SVP
SVP in flc-3
SVP in FLC
[3-5]
[3-5]
[3-10]
[3-10]
AT2G1830
Region 12
[3-15]
[3-15]
[3-15]
[3-15]
AT2G25460

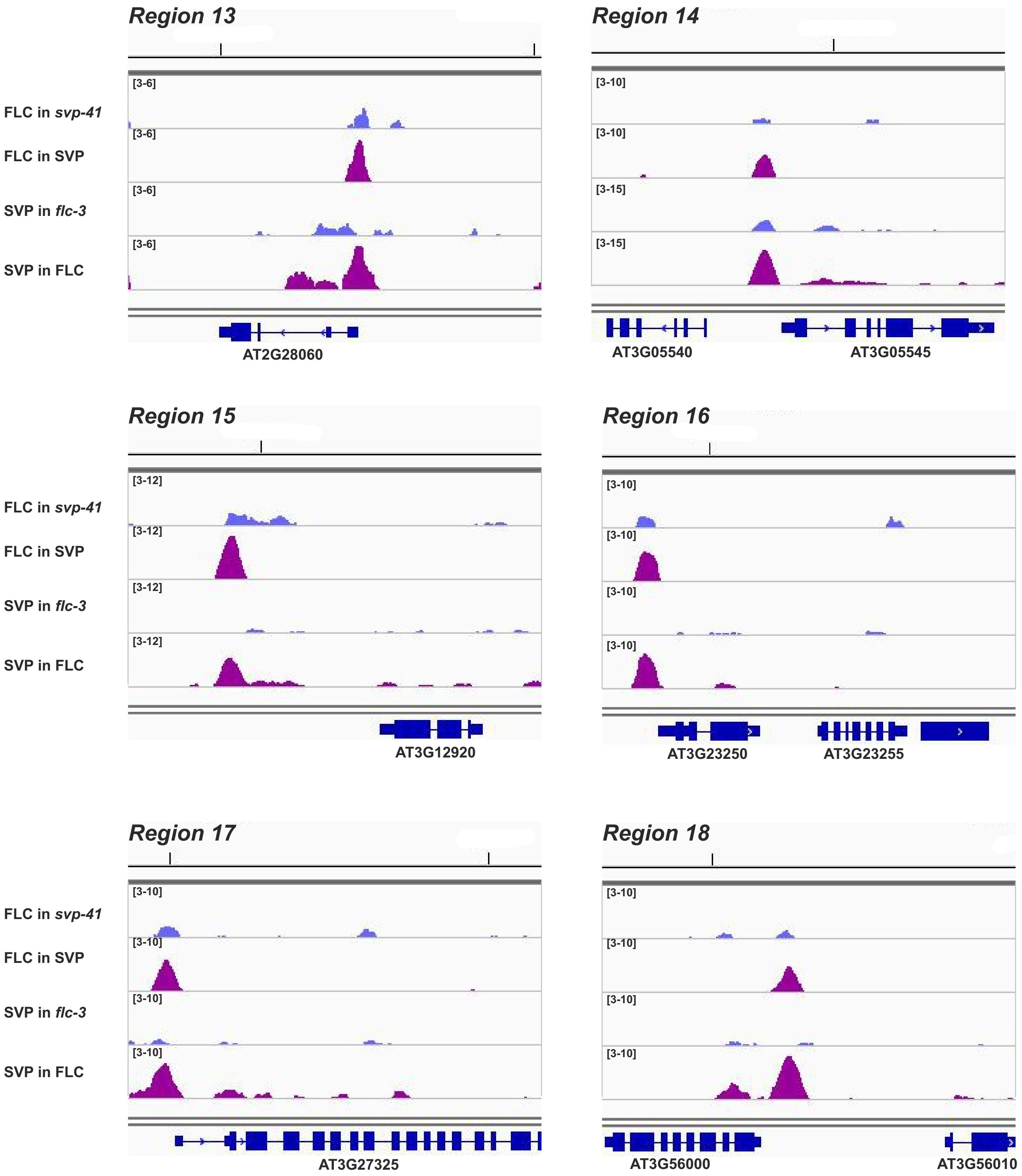
Region 13
Region 14
Region 15
Region 16
Region 17
Region 18
FLC in svp-41
FLC in SVP
SVP in flc-3
SVP in FLC
[3-6]
[3-10]
[3-15]
[3-12]
AT2G28060
AT3G05540
AT3G05545
AT3G12920
AT3G23250
AT3G23255
AT3G27325
AT3G56000
AT3G56010

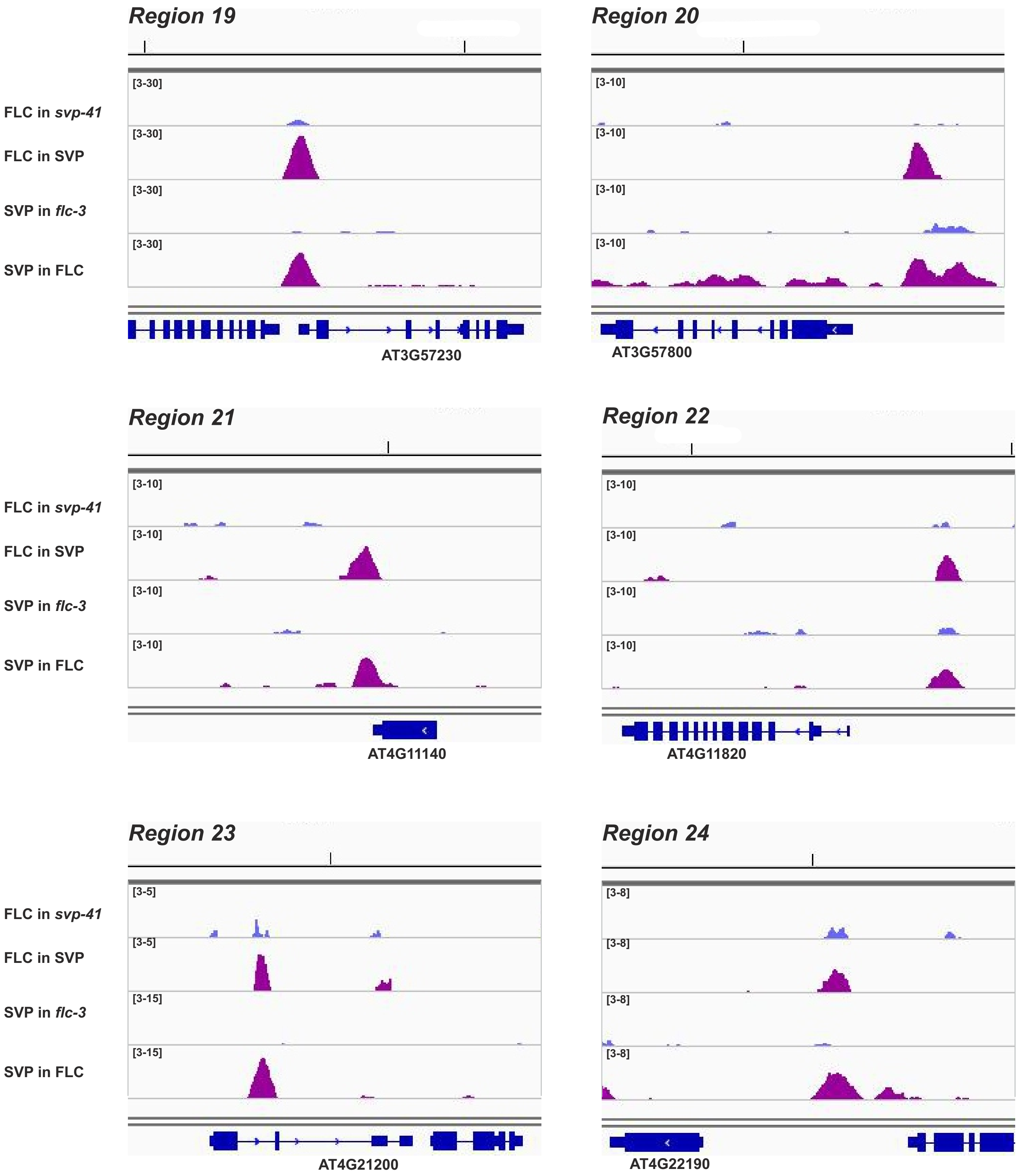

Region 19
FLC in *svp-41*
[3-30]
FLC in SVP
[3-30]
SVP in *flc-3*
[3-30]
SVP in FLC
[3-30]
AT3G57230
Region 20
[3-10]
[3-10]
[3-10]
[3-10]
AT3G57800
Region 21
FLC in *svp-41*
[3-10]
FLC in SVP
[3-10]
SVP in *flc-3*
[3-10]
SVP in FLC
[3-10]
AT4G11140
Region 22
[3-10]
[3-10]
[3-10]
[3-10]
AT4G11820
Region 23
FLC in *svp-41*
[3-5]
FLC in SVP
[3-5]
SVP in *flc-3*
[3-15]
SVP in FLC
[3-15]
AT4G21200
Region 24
[3-8]
[3-8]
[3-8]
[3-8]
AT4G22190

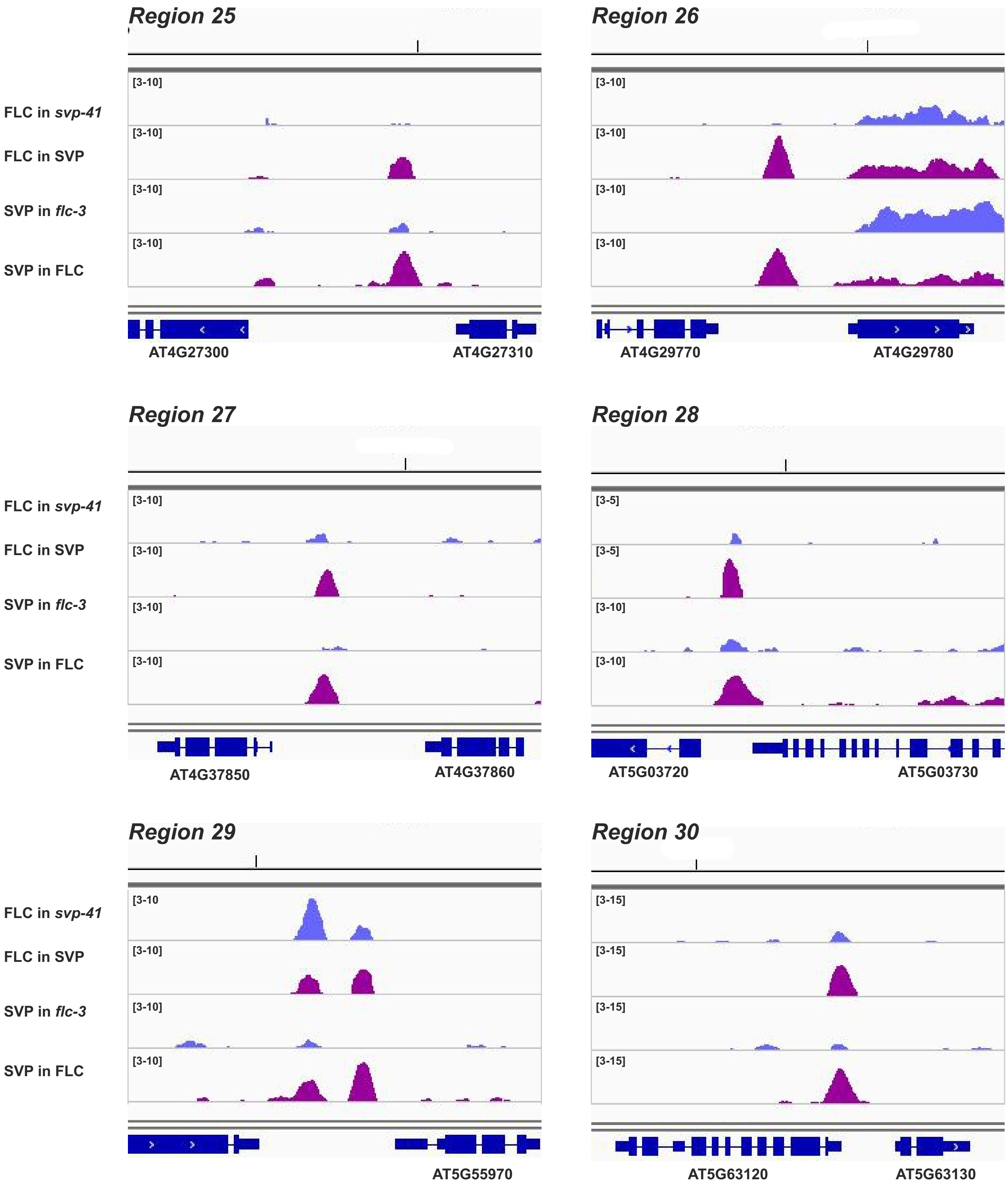
Region 25
FLC in svp-41
FLC in SVP
SVP in flc-3
SVP in FLC
[3-10]
[3-10]
[3-10]
[3-10]
AT4G27300
AT4G27310
Region 26
[3-10]
[3-10]
[3-10]
[3-10]
AT4G29770
AT4G29780
Region 27
FLC in svp-41
FLC in SVP
SVP in flc-3
SVP in FLC
[3-10]
[3-10]
[3-10]
[3-10]
AT4G37850
AT4G37860
Region 28
[3-5]
[3-5]
[3-10]
[3-10]
AT5G03720
AT5G03730
Region 29
FLC in svp-41
FLC in SVP
SVP in flc-3
SVP in FLC
[3-10
[3-10]
[3-10]
[3-10]
AT5G55970
Region 30
[3-15]
[3-15]
[3-15]
[3-15]
AT5G63120
AT5G63130

Region	Peak Location	Associated Genes
Region 1	Chr1:1588052-1588318	**AT1G05410** Protein of unknown function (DUF1423),<br>**AT1G05420** ovate family protein 12 (OFP12)
Region 2		**AT1G07128** NA<br>**AT1G07135** glycine-rich protein
Region 3		**AT1G12610** DWARF AND DELAYED FLOWERING 1 (DDF1)
Region 4		**AT1G14890** Plant invertase/pectin methylesterase inhibitor superfamily protein<br>**AT1G14900** high mobility group A (HMGA)
Region 5	Chr1:5945181-5945641	**AT1G17350** NADH:ubiquinone oxidoreductase intermediate-associated protein 30,<br>**AT1G17360** BEST Arabidopsis thaliana protein match is: COP1-interacting protein-related
Region 6	Chr1:8431888-8432297	**AT1G23860** RS-containing zinc finger protein 21 (RSZP21),<br>**AT1G23870** trehalose-phosphatase/synthase 9 (TPS9)
Region 7	Chr1:8983623-8983948	**AT1G25560** TEMPRANILLO 1 (TEM1),
Region 8	Chr1:19513031-19513330	**AT1G52390** unknown protein<br>**AT1G52400** beta glucosidase 18 (BGLU18)
Region 9		**AT1G52890** NAC domain containing protein 19 (NAC019)
Region 10		**AT1G71696** SUPPRESSOR OF LLP1 1 (SOL1)<br>**AT1G71697** choline kinase 1 (CK1)
Region 11	Chr2:368825-369022	**AT2G01830** WOODEN LEG (WOL),
Region 12	Chr2:10836136-10836667	**AT2G25460** CONTAINS InterPro DOMAIN/s: C2 calcium-dependent membrane targeting<br>**AT2G25470** receptor like protein 21 (RLP21)
Region 13	Chr2:11952075-11952335	**AT2G28060** 5'-AMP-activated protein kinase beta-2 subunit protein,
Region 14		**AT3G05540** Methionine sulfoxide reductase (MSS4-like) family protein<br>**AT3G05545** RING/U-box superfamily protein
Region 15	Chr3:4119358-4119728	NA
Region 16	Chr3:8308906-8309182	**AT3G23250** MYB15,<br>**AT3G2325**5 unknown protein
Region 17	Chr3:10094742-10095088	**AT3G27325** hydrolases, acting on ester bonds,
Region 18	Chr3:20786142-20786287	**AT3G56000** cellulose synthase like A14 (CSLA14),<br>**AT3G56010** unknown protein
Region 19		**AT3G57230** AGAMOUS-LIKE 16
Region 20		**AT3G57800** bHLH DNA-binding superfamily protein
Region 21	Chr4:6794561-6794843	**AT4G11140** cytokinin response factor 1 (CRF1),
Region 22		**AT4G11820** HYDROXYMETHYLGLUTARYL-COA SYNTHASE (HMGS)
Region 23		**AT4G21200** GIBBERELLIN 2-OXIDASE 8
Region 24		**AT4G22190** unknown protein
Region 25	Chr4:13674604-13674936	**AT4G27300** S-locus lectin protein kinase family protein<br>**AT4G27310** B-box type zinc finger family protein
Region 26	Chr4:14578403-14578760	**AT4G29770** unknown protein<br>**AT4G29780** unknown protein
Region 27	Chr4:17798605-17798904	**AT4G37850** bHLH DNA-binding superfamily protein
Region 28		**AT5G03720** heat shock transcription factor A3 (HSFA3)<br>**AT5G03730** CONSTITUTIVE TRIPLE RESPONSE 1 (CTR1)
Region 29		**AT5G55970** RING/U-box superfamily protein
Region 30	Chr5:25322058-25322490	**AT5G63120** P-loop containing nucleoside triphosphate hydrolases superfamily protein<br>**AT5G63130** Octicosapeptide/Phox/Bem1p family protein
