# Supplementary material for: Combinatorial activities of SHORT VEGETATIVE PHASE and FLOWERING LOCUS C define distinct modes of flowering regulation in Arabidopsis
Source: Genome Biol. 2015 Feb 11;16(1):31. doi: 10.1186/s13059-015-0597-1 (PMC4378019; doi:10.1186/s13059-015-0597-1)
Supplement: Additional file 28: Figure S18. — Venn diagrams showing the overlapping set of putative targets for SVP and FLC between this and previous studies. (A) Venn diagram for SVP ChIP-seq targets in Gregis et al. [4], Tao et al. [9] and the results presented in this study. (B) Venn diagram for targets of FLC reported in Deng et al. [3] and the results presented in this study. (C) Comparison between experimental procedures, sequencing technologies, and bioinformatic approaches used for genome-wide identification of SVP and FLC targets in the studies in (A) and (B). [file 13059_2015_597_MOESM28_ESM.pdf]

A

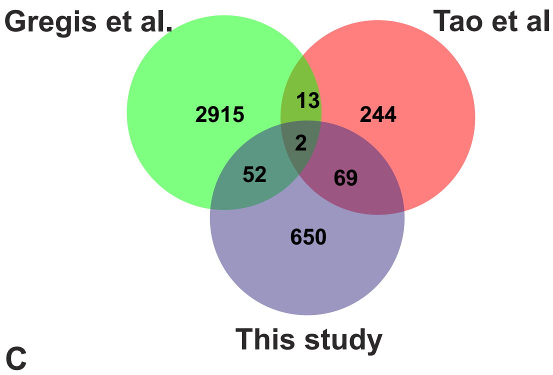

B

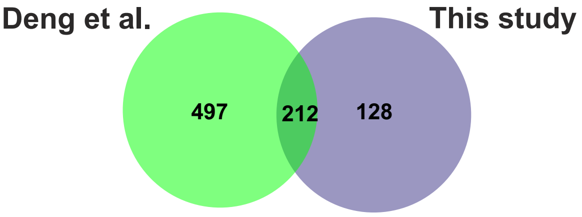

C

SVP ChIP	Tao et al.	Gregis et al.	This study
**Technology**	ChIP-chip	Genome AnalyzerII	Illumina HiSeq 2000
**Genotype**	*35S:SVP*	pSVP:SVP:GFP	pSVP:SVP:GFP
**Growth conditions**	9 days LD	2 weeks SD	2 weeks SD
**Antibody**	anti-SVP	anti-GFP (Clontech)	anti-GFP (abcam)
**Replicates n°**	3	2(1)*	3
**Mapping Tools**	-	SeqMan	Bowtie
**Analysis Tools (Peak caller)**	TileMapv2	CSAR	MACS (v2) and NarrowPeaks

* 2 biological replicates were used for DNA preparation, but reads from each replicate were pooled before read mapping for analysis.

FLC ChIP	Deng et al.	This study
**Technology**	Genome Analyzer II	Illumina HiSeq 2000
**Genotype**	*FRI FLC SVP*	*FRI FLC SVP*
**Growth conditions**	12 days L D	2 weeks SD
**Antibody**	anti-FLC	anti-FLC
**Replicates n°**	1	3
**Mapping Tools**	not reported	Bowtie
**Analysis Tools (Peak caller)**	QuEST and MACS	MACS (v2) and NarrowPeaks
